# Health insurance coverage among incident cancer cases from population-based cancer registries in 49 US states, 2010–2019

**DOI:** 10.1093/haschl/qxad083

**Published:** 2024-01-11

**Authors:** Xin Hu, Nuo Nova Yang, Qinjin Fan, K Robin Yabroff, Xuesong Han

**Affiliations:** Department of Public Health Sciences, University of Virginia School of Medicine, Charlottesville, VA 22911, United States; Surveillance and Health Equity Science, American Cancer Society, Atlanta, GA 30144, United States; Surveillance and Health Equity Science, American Cancer Society, Atlanta, GA 30144, United States; Surveillance and Health Equity Science, American Cancer Society, Atlanta, GA 30144, United States; Surveillance and Health Equity Science, American Cancer Society, Atlanta, GA 30144, United States

**Keywords:** health insurance coverage, cancer, Medicaid expansion

## Abstract

Having health insurance coverage is a strong determinant of cancer care access and survival in the United States. The expansion of Medicaid income eligibility under the Affordable Care Act has increased insurance coverage for working-age adults. Using data from the Cancer Incidence in North America (CiNA) in 2010–2019, we identified 6 432 117 incident cancer cases with known insurance status diagnosed at age 18–64 years from population-based registries of 49 states. Considerable variation in Medicaid coverage and uninsured rate exists across states, especially by Medicaid expansion status. Among expansion states, Medicaid coverage increased from 14.1% in 2010 to 19.9% in 2019, while the Medicaid coverage rate remained lower (range = 11.7% – 12.7%) in non-expansion states. The uninsured rate decreased from 4.9% to 2.1% in expansion states, while in non-expansion states, the uninsured rate decreased slightly from 9.5% to 8.1%. In 2019, 111 393 cancer cases (16.9%) had Medicaid coverage at diagnosis (range = 7.6%–37.9% across states), and 48 357 (4.4%) were uninsured (range = 0.5%–13.2%). These estimates suggest that many patients with cancer may face challenges with care access and continuity, especially following the unwinding of COVID-19 pandemic protections for Medicaid coverage. State cancer prevention and control efforts are needed to mitigate cancer care disparities among vulnerable populations.

## Introduction

Cancer is a leading cause of death for working-age Americans.^1^ Health insurance coverage is a key determinant of cancer care access and outcomes.^2^ Lack of health insurance underlies many longstanding disparities in cancer survival and mortality. For people with low incomes or from socioeconomically and/or medically disadvantaged backgrounds, Medicaid can help ensure accessible and quality cancer care that may not otherwise be affordable for them.^3^ Health policies that increase coverage options, such as the expansion of Medicaid income eligibility under the Affordable Care Act (ACA), have led to historic gains in coverage in both the general population and individuals diagnosed with cancer^4-6^ and are associated with increased cancer screening, earlier stage at diagnosis, better cancer care quality and survival, as well as decreased cancer disparities.^7,8^ However, 10 states have not expanded Medicaid as of September 2023,^9^ potentially widening geographic disparities in access to cancer care and patient outcomes. Proposals for Medicaid work requirements and the unwinding of COVID-19 pandemic protections for continuous Medicaid coverage threaten recent progress. The Congressional Budget Office estimated that approximately 15.5 million low-income individuals would lose Medicaid coverage in 2023.^10^ As of October 11, 2023, at least 8.7 million individuals were disenrolled from Medicaid, many for procedural reasons.^11^ Disenrollment rate varied widely by state, from 66% in Texas to 11% in Illinois.^11^

Existing estimates of patients with cancer covered under Medicaid and other insurance programs were mostly based on selected geographic regions (eg, 13 states included in the Surveillance, Epidemiology, and End Results [SEER] program) or subsamples (ie, hospital registries or survey samples), or only a short period of follow-up after Medicaid expansion under the ACA.^3,12,13^ State-level estimates, which are essential for state and local health policy and program implementation, are rarely available. Moreover, to better understand the challenges posed by the recent unwinding of COVID-19 protections for Medicaid enrollees diagnosed with cancer, a population that faces substantial financial burden from cancer care,^7^ understanding how Medicaid coverage for individuals with cancer has changed in recent years is critical.

Using population-based cancer registry data from 48 states and the District of Columbia in 2010–2019, we identified 6 432 117 newly diagnosed cancer cases aged 18–64 years with known insurance status. This study aims to describe (1) the trend in Medicaid coverage, other insurance coverage, and uninsured rate by states' expansion status, and for individual states, and (2) the number of cancer cases covered by Medicaid, other insurance, and were uninsured in the most recent year of data available (ie, 2019) overall and by key sociodemographic characteristics such as age group, sex, and race/ethnicity. These findings can inform state efforts in providing equitable cancer prevention and control.

## Data and methods

### Data

We used the Cancer Incidence in North America (CiNA) incidence dataset 2010–2019 compiled by the North American Association of Central Cancer Registries (NAACCR).^14^ The CiNA data are evaluated for accuracy and certified by NAACCR's high-quality data standards every year, and have been widely used in cancer surveillance.^15^ We identified incident cancer cases diagnosed at ages 18–64 years from 48 states and the District of Columbia who agreed to participate in the study (Table S1). Kansas and Minnesota were not included due to lack of consent from the state registry or data not meeting NAACCR quality standards. Data for Nevada were available in 2010–2014.

We grouped states into 3 categories: states that adopted and implemented Medicaid expansion by 2014 (*n* = 26), late-expansion states (expansion between 2015 and 2019; *n* = 7), and non-expansion states as of 2019 (*n* = 16) (Table S1). We excluded cancer cases with unknown insurance status at diagnosis from the primary analysis (*n* = 677 499; Figure S1) and present results including cancer cases with unknown insurance status in Figures S3–S5 and Table S3.

### Outcomes and statistical analysis

The study outcome was primary insurance coverage type at diagnosis, which was classfied into Medicaid, other insurance, and uninsured. We examined trends of the annual percentage of incident cancer cases with Medicaid coverage and uninsured at diagnosis by expansion status, and for each individual state from 2010 to 2019. We described the number and percentage of three insurance coverage types by age group, sex, and race/ethnicity among cancer incident cases in 2019 overall and for each state. We also presented maps showing the state variation in Medicaid coverage and uninsured rate in 2019. Logistic regression was used to test differential changes from 2010 to 2019 by expansion status, using an interaction term between year of diagnosis and expansion status. Data were exported from the SEER*Stat 8.4.0.1 (National Cancer Institute & Information Management Services, Inc). We used Microsoft Excel (Microsoft Corporation) and ArcMap version 10.8.2 (ESRI) for data management and visualization and SAS software version 9.4 (SAS Institute) for statistical analysis. Statistical significance was determined using 2-sided tests with *P* < .05.

## Results

**Figure 1.**
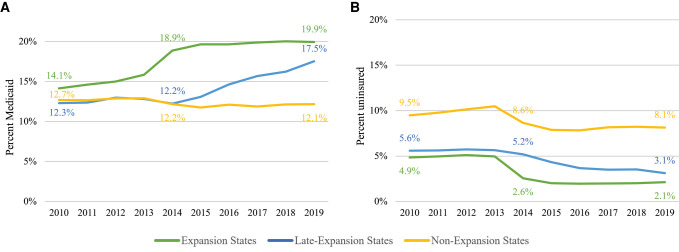
Trend in the percentage of Medicaid insured and uninsured by expansion status among individuals aged 18–64 years newly diagnosed with cancer, 2010–2019. Source: Authors' analysis of Cancer Incidence in North America (CiNA) 2010–2019 compiled by the North American Association of Central Cancer Registries. (A) Medicaid. (B) Uninsured. Individual states' status in Medicaid expansion can be found in Table S1. Cases with unknown insurance status at diagnosis were excluded.

The analytical sample included 6 432 117 cancer incident cases aged 18–64 years diagnosed in 2010–2019, including 993 695 with Medicaid and 340 055 who were uninsured at diagnosis. Among 3 448 747 cancer incident cases diagnosed in expansion states, the percentage with Medicaid increased immediately after the implementation of the ACA by 3.0% points (ppts), from 15.8% in 2013 to 18.9% in 2014, and continued increasing to 19.9% in 2019. Uninsured rates among incident cancer cases decreased by 2.4 ppts from 5.0% in 2013 to 2.6% in 2014 and stabilized at 2.0% from 2015 to 2019 (Figure 1). There were consistent trends of increases in Medicaid coverage after 2014 by almost all individual states (Figure S2). States that experienced the largest increase in Medicaid coverage among incident cancer cases in 2014 included Oregon (8.1 ppts), New Mexico (7.5 ppts), and Kentucky (7.4 ppts). The largest decrease in uninsured rate was also seen in Oregon (−5.2 ppts), New Mexico (−4.2 ppts), and Kentucky (−5.5 ppts) (Figure S2).

Among 822 043 cancer incident cases diagnosed in the 7 late-expansion states, 5 states (Alaska, Indiana, Louisiana, Montana, and Pennsylvania) expanded by mid-2016 and 2 states (Maine and Virginia) expanded in early 2019. Overall, the percentage of cancer cases with Medicaid increased immediately following states' corresponding implementation of expansion (Figure 1) —for example, from 10.0% to 16.3% for Alaska and 12.3% to 14.0% for Pennsylvania after expansion in 2015 (Figure S2). The uninsured rate among incident cancer cases also showed immediate decreases—from 5.0% to 4.3% for Alaska and 1.8% to 1.5% for Pennsylvania after expansion in 2015. Across individual states, Montana (expanded Medicaid eligibility on January 1, 2016) had the largest increase in Medicaid coverage (13.6 ppts) and the largest decrease in uninsured rate (−5.9 ppts) among incident cancer cases from 2010–2019 (Figure S2).

Among 2 161 327 cancer incident cases diagnosed in non-expansion states, the percentage of cancer cases with Medicaid remained stable from 12.7% in 2010 to 12.1% in 2019, for a total of 266 086 cancer cases with Medicaid coverage at diagnosis in all years. There was a slight decrease in uninsured rate from 9.5% to 8.1% in 2010–2019, 190 533 cancer cases across the years (Figure 1). Across individual states, 9 states had increases in Medicaid coverage, ranging from 0.1 ppt (Missouri) to 6.2 ppts (North Carolina) among incident cancer cases. Uninsured rates decreased in most states, ranging from −0.04 ppts in Georgia to −2.7 ppts in South Carolina, but not in North Carolina (1.2 ppts) and Idaho (0.7 ppts, Figure S2).

The changes in the percentage of incident cancer cases covered by Medicaid from 2010 to 2019 in expansion states (5.8 ppts, from 14.1% to 19.9%) and late-expansion states (5.2 ppts, from 12.3% to 17.5%) were statistically significantly different from non-expansion states (−0.6 ppt, from 12.7% to 12.1%, *P* values < .001). The changes in uninsured rate were also significantly different comparing expansion states (−2.7 ppts, *P* < .001) and late-expansion states (−2.5 ppts, *P* = .019) with non-expansion states (−1.3 ppts).

**Figure 2.**
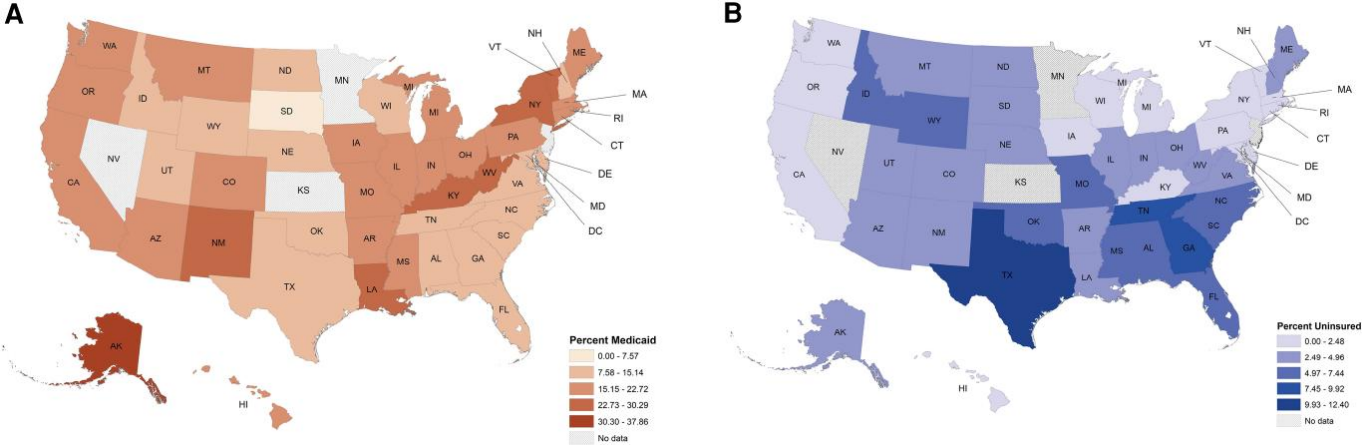
The percentage of Medicaid insured and uninsured among individuals aged 18–64 years newly diagnosed with cancer. Source: Authors' analysis of Cancer Incidence in North America (CiNA) 2010–2019 compiled by the North American Association of Central Cancer Registries. (A) Medicaid. (B) Uninsured. Percentages were calculated excluding cancer cases with unknown insurance status.

In 2019, a total of 708 403 new cancer cases were daignosed in the United States with known insurance status. Among them, 16.9% were covered by Medicaid (*n* = 111 393) and 78.7% by other insurance (*n* = 519 730), leaving 4.4% (*n* = 28 923) without any insurance coverage at diagnosis. The percentage of cancer cases with Medicaid at diagnosis was highest in Alaska (24.1%), Kentucky (26.0%), Louisiana (27.6%), New York (24.1%), New Mexico (25.5%), Vermont (23.3%), and West Virginia (24.2%). States that had the highest number of cancer cases with Medicaid coverage at diagnosis were California (*n* = 15 781) and New York (*n* = 11 424). Texas had the highest uninsured rate among all states (13.2%), followed by Georgia (9.9%) and Tennessee (8.4%). On the other hand, Massachusetts (0.5%), Hawaii (0.9%), District of Columbia (1.0%), and New York (1.2%) had the lowest uninsured rate among incident cancer cases (Figure 2 and Table S2). States with the largest number of uninsured cancer cases at diagnosis were Texas (*n* = 6890), Florida (*n* = 2788), and Georgia (*n* = 2137).

**Figure 3.**
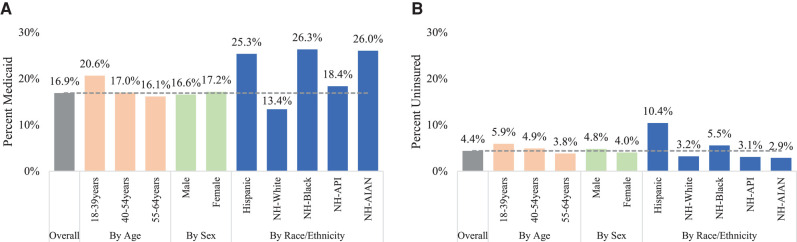
The percentage with Medicaid coverage and uninsured among individuals aged 18–64 years newly diagnosed with cancer by age, sex, and race/ethnicity across states, 2019. Source: Authors' analysis of Cancer Incidence in North America (CiNA) 2010–2019 compiled by the North American Association of Central Cancer Registries. (A) Medicaid. (B) Uninsured. Percentages were calculated excluding cancer cases with unknown insurance status. Abbreviations: AIAN, American Indian and Alaska Native; API, Asian Pacific Islander; NH, non-Hispanic.

There was also variation across sociodemographic subgroups. Incident cancer cases diagnosed at ages 18–39 years had both the highest percentage of Medicaid coverage (20.6%) and being uninsured (5.9%) compared with those diagnosed at ages 40–54 and 55–64 years. The percentage of Medicaid coverage was higher among Hispanic (25.4%), non-Hispanic Black (26.4%), non-Hispanic Asian Pacific Islander (18.4%), and non-Hispanic American Indian and Alaska Native (26.6%), compared with non-Hispanic White (13.4%). Hispanic (10.4%) and non-Hispanic Black (5.5%) also had higher uninsured rates compared with non-Hispanic White (3.2%) individuals diagnosed with cancer (Figure 3). Compared with 2010, while increases in Medicaid coverage were observed across all sociodemographic subgroups, differences in Medicaid coverage between male and female cancer cases became smaller (10.7% vs. 13.1% in 2010, 16.6% vs. 17.2% in 2019); the largest gains were observed among the age group of 55–64 years (10.2% in 2010 vs 16.1% in 2019), non-Hispanic Blacks (20.0% in 2010 vs. 26.3% in 2019), and non-Hispanic American Indians and Alaska Natives (18.9% in 2010 vs. 26.0% in 2019, Figure S6, Figure 3).

Consistent with patterns in our national-level analysis, across individual states, a higher percentage of Medicaid coverage among incident cancer cases was observed in younger age groups (18–39 years), and among racial minority groups such as Hispanics and non-Hispanic Blacks compared with non-Hispanic Whites (Table S2). Analyses including cancer cases with unknown insurance status at diagnosis showed similar patterns (Figures S3–S5 and Table S3).

## Limitations

Our study has limitations. First, some cancer cases had unknown insurance status. Although analyses including unknown insurance status showed similar patterns, the absolute numbers of cancer cases by insurance type are understated. Further examination of the cancer cases with unknown insurance status found that they tend to be older, male, and reported as non-Hispanic Black or other or unknown racial/ethnic groups. The proportion of Medicaid coverage was lower in these subgroups among those with known insurance status (Table S4). Greater completeness in payer information collection for cancer registries is warranted to more precisely evaluate the Medicaid coverage rate and uninsured rate for states. Second, the insurance information was only collected following diagnosis. In some states, uninsured individuals can gain Medicaid retrospectively due to cancer diagnosis^16^; thus, the Medicaid coverage we reported at the exact time of diagnosis may be overestimated. On the other hand, coverage continuity after cancer diagnosis is also critical for health outcomes.^17^ We were unable to assess insurance coverage continuity throughout the cancer treatment and survivorship in this study due to lack of information; future research with more comprehensive information (eg, cancer registry data linked to all payer claims data) is warranted.

## Discussion

Using a recent nationwide population-based sample of incident cancer cases, we observed a considerable increase in Medicaid coverage and a decrease in uninsured rates in states that expanded Medicaid income eligibility under the ACA. Medicaid coverage changed little in non-expansion states, and uninsured rates remained relatively high, with 19 136 newly diagnosed cancer cases without health insurance coverage in 2019 alone, and 111 040 cases during 2014–2019.

Our estimates contribute to the literature in several ways. First, our analysis included states that are not in the SEER program—therefore, with increased national representativeness. Given the population-based nature of the data, these estimates are not subject to survey response biases, and more accurately reflect the sociodemographic composition of the state and the nation. Last, we added more recent estimates up to 2019.

Prior research found that Medicaid expansion was associated with reductions in racial disparities in health insurance coverage, early stage diagnosis, and survival.^18,19^ The variation we observed in insurance coverage by race and ethnicity may contribute to disparities in subsequent survival. With the most recent adoption of Medicaid expansion by South Dakota and North Carolina, there are still 10 states that have not expanded Medicaid as of September 2023.^9^ Given overwhelming evidence of the effectiveness of Medicaid expansion in improving health outcomes,^20^ continuing efforts towards more Medicaid expansions are warranted.

We quantified substantial state variations in insurance status of patients newly diagnosed with cancer, a costly life-threatening disease. Many state-level characteristics could factor in these variations, including state Medicaid expansion status and specific provisions (eg, income eligibility thresholds, work requirements), income distribution of the population, local employers' provision of health insurace (the primary source of private insurance coverage), and state-specific insurance programs and cancer programs. In general, states with the highest Medicaid coverage rate are predominantly states that adopted Medicaid expansion, which could also effectively reduce uninsured rates. For example, two Medicaid expansion states – New York and California had relatively high Medicaid coverage (24.1% and 22.0%, respectively) and low uninsured rates (1.2% and 1.3%, respectively). These 2 populous states also had the largest number of cancer cases covered by Medicaid (11 424 and 15 781, respectively). In contrast, a non-expansion state, Texas, had the highest number of incident cases in 2019 (*n* = 55 565),^21^ the highest number of uninsured cancer cases (*n* = 6890), and the highest uninsured rate (13.2%). Other non-expansion states such as Florida and Georgia also had large numbers of uninsured cancer cases (2788 and 2137, respectively). Other state-level policies could also affect Medicaid coverage and uninsured rates. In Massachusetts, where health care reform efforts started in 2006, the uninsured rate is the lowest (0.5%) among all states. Other cancer-specific programs include the National Breast and Cervical Cancer Early Detection Program and the Breast and Cervical Cancer Treatment Program, which allow states the flexibility to provide more generous Medicaid coverage for breast and cervical cancer screening, diagnosis, and treatment through modifying the age and income eligibility thresholds.^22,23^ Some states, such as New York, further expanded their cancer program to cover additional cancer sites such as prostate and colorectal cancers.^24^ As health insurance is crucial to ensure timely access to high-quality cancer care, patients residing in non-Medicaid expansion states or states with less generous insurance program provision may lack affordable health care and have worse health outcomes. Adequate state budgets for Medicaid and other insurance programs are critical to ensure access to timely, high-quality cancer care.

In conclusion, this study provides population-based estimates of Medicaid coverage and uninsurance in newly diagnosed cancer cases in the United States, which can inform future efforts to implement state-level Medicaid expansion and other cancer prevention and control programs, state health care budget planning, and efforts to reduce disparities among vulnerable populations.

## Supplementary Material

qxad083_Supplementary_Data

## Data Availability

The data underlying this article were provided by the American Association of Central Cancer Registries (NAACCR) by permission. The data cannot be shared publicly per the Data Use Agreement. The NAACCR CiNA Public Use Data Set with a limited number of variables is available through application at https://www.naaccr.org/cina-public-use-data-set/.
