# Supplementary material for: Health insurance coverage among incident cancer cases from population-based cancer registries in 49 US states, 2010–2019
Source: Health Aff Sch. 2024 Jan 11;2(1):qxad083. doi: 10.1093/haschl/qxad083 (PMC10986217; doi:10.1093/haschl/qxad083)
Supplement: qxad083_Supplementary_Data [file qxad083_Supplementary_Data.zip › Appendix_InsuranceDataBrief_IncludeUnknown_11-2-2023.docx.docx]

**Supplemental Material**

Table S1. States by Medicaid expansion status as of 2019

| **Expansion states (26)** | Arizona, Arkansas, California, Colorado, Connecticut, Delaware, District of Columbia, Hawaii, Illinois, Iowa, Kentucky, Maryland, Massachusetts, Michigan, Nevada, New Hampshire, New Jersey, New Mexico, New York, North Dakota, Ohio, Oregon, Rhode Island, Vermont, Washington, and West Virginia |
| --- | --- |
| **Late Expansion states (7)** | Alaska, Indiana, Louisiana, Maine, Montana, Pennsylvania, and Virginia |
| **Non-expansion states (16)** | Alabama, Florida, Georgia, Idaho, Mississippi, Missouri, Nebraska, North Carolina, Oklahoma, South Carolina, South Dakota, Tennessee, Texas, Utah, Wisconsin, and Wyoming |

Figure S1. Sample Derivation Process


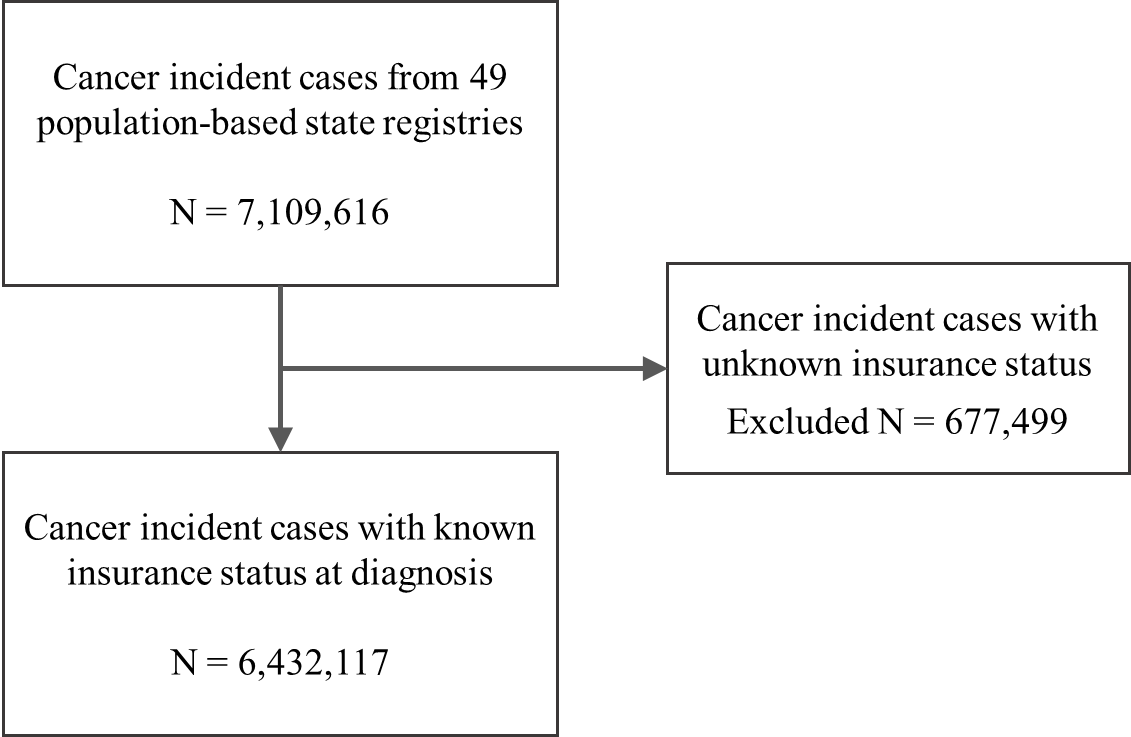


Figure S2. Trend of Percent Medicaid Insured and Uninsured By States Among Incident Cancer Cases Diagnosed at Ages 18-64 Years in 2010-2019, Excluding Cases With Unknown Insurance Status

1. Expansion States
2. Late Expansion States
3. Non-Expansion States


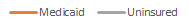


Source: Authors’ analysis of Cancer Incidence in North America (CiNA) 2010-2019 compiled by the North American Association of Central Cancer Registries

Notes: Cases with unknown insurance status at diagnosis were excluded.

Table S2. Insurance Coverage Among Incident Cancer Cases Diagnosed At Ages 18-64 Years by Age, Sex, Race/Ethnicity Across States in 2019, Excluding Cases With Unknown Insurance Status

|  |  | **Overall** | **18-39years** | **40-54years** | **55-64years** | **Male** | **Female** | **Hispanic** | **NH-White** | **NH-Black** | **NH-API** | **NH-AIAN** |
| --- | --- | --- | --- | --- | --- | --- | --- | --- | --- | --- | --- | --- |
| US | Total | 708,403 | 74,146 | 224,911 | 409,346 | 331,008 | 377,395 | 81,513 | 484,126 | 93,926 | 30,345 | 4,084 |
|  | Medicaid | 111,393 (16.9%) | 14,284 (20.6%) | 35,751 (17.0%) | 61,358 (16.1%) | 50,387 (16.6%) | 61,006 (17.2%) | 19,607 (25.4%) | 60,887 (13.4%) | 23,231 (26.4%) | 5,361 (18.4%) | 1,037 (26.6%) |
|  | Other Insured | 519,730 (78.7%) | 50,813 (73.4%) | 164,518 (78.1%) | 304,399 (80.1%) | 239,421 (78.6%) | 280,309 (78.8%) | 49,670 (64.2%) | 378,876 (83.4%) | 60,036 (68.1%) | 22,830 (78.5%) | 2,747 (70.5%) |
|  | Uninsured | 28,923 (4.4%) | 4,084  (5.9%) | 10,341 (4.9%) | 14,498 (3.8%) | 14,619 (4.8%) | 14,304 (4.0%) | 8,056 (10.4%) | 14,613 (3.2%) | 4,889 (5.5%) | 901  (3.1%) | 114 (2.9%) |
|  |  |  |  |  |  |  |  |  |  |  |  |  |
| Alabama | Total | 11,913 | 1,092 | 3,791 | 7,030 | 5,821 | 6,092 | 179 | 8,254 | 3,076 | 113 | <11 |
|  | Medicaid | 1,428 (13.2%) | 167  (17.0%) | 458 (13.2%) | 803 (12.6%) | 612 (11.7%) | 816 (14.6%) | 21 (12.7%) | 794 (10.4%) | 598 (20.8%) | 11 (10.9%) | <11 |
|  | Other Insured | 8,587 (79.3%) | 704  (71.5%) | 2,714 (78.4%) | 5,169 (81.1%) | 4,145 (79.4%) | 4,442 (79.3%) | 90 (54.5%) | 6,361 (83.2%) | 2,025 (70.4%) | 82 (81.2%) | <11 |
|  | Uninsured | 810 (7.5%) | 114  (11.6%) | 291  (8.4%) | 405  (6.4%) | 466 (8.9%) | 344 (6.1%) | 54 (32.7%) | 486 (6.4%) | 253 (8.8%) | <11 | <11 |
|  |  |  |  |  |  |  |  |  |  |  |  |  |
| Alaska | Total | 1,478 | 166 | 462 | 850 | 654 | 824 | 36 | 1,033 | 41 | 97 | 260 |
|  | Medicaid | 320 (24.1%) | 37 (25.5%) | 94 (22.6%) | 189 (24.7%) | 143 (24.9%) | 177 (23.5%) | <11 | 186 (20.5%) | 12 (30.0%) | 25 (28.4%) | 87 (33.9%) |
|  | Other Insured | 958 (72.2%) | 98 (67.6%) | 307 (73.8%) | 553 (72.2%) | 407 (70.9%) | 551 (73.2%) | 20 (60.6%) | 687 (75.7%) | 27 (67.5%) | 55 (62.5%) | 168 (65.4%) |
|  | Uninsured | 49 (3.7%) | <11 | 15 (3.6%) | 24 (3.1%) | 24 (4.2%) | 25 (3.3%) | <11 | 35 (3.9%) | <11 | <11 | <11 |
|  |  |  |  |  |  |  |  |  |  |  |  |  |
| Arizona | Total | 12,448 | 1,432 | 3,956 | 7,060 | 5,616 | 6,832 | 2,665 | 8,336 | 523 | 294 | 293 |
|  | Medicaid | 2,363 (20.5%) | 350 (26.0%) | 783 (21.3%) | 1,230 (18.9%) | 1,081 (21.0%) | 1,282 (20.1%) | 741 (28.9%) | 1,262 (16.4%) | 152 (29.9%) | 52 (18.1%) | 119 (43.1%) |
|  | Other Insured | 8,696 (75.5%) | 931 (69.2%) | 2,717 (73.9%) | 5,048 (77.7%) | 3,840 (74.8%) | 4,856 (76.1%) | 1,585 (61.8%) | 6,264 (81.3%) | 338 (66.4%) | 228 (79.2%) | 156 (56.5%) |
|  | Uninsured | 457 (4.0%) | 64 (4.8%) | 177 (4.8%) | 216 (3.3%) | 215 (4.2%) | 242 (3.8%) | 238 (9.3%) | 183 (2.4%) | 19 (3.7%) | <11 | <11 |
|  |  |  |  |  |  |  |  |  |  |  |  |  |
| Arkansas | Total | 7,512 | 719 | 2,298 | 4,495 | 3,665 | 3,847 | 224 | 5,888 | 1,151 | 70 | 32 |
|  | Medicaid | 1,076 (15.8%) | 130 (20.3%) | 330 (15.9%) | 616 (15.1%) | 512 (15.3%) | 564 (16.3%) | 18 (9.3%) | 775 (14.4%) | 265 (24.1%) | <11 | <11 |
|  | Other Insured | 5,507 (80.9%) | 482 (75.4%) | 1,680 (80.7%) | 3,345 (81.8%) | 2,712 (81.1%) | 2,795 (80.7%) | 144 (74.6%) | 4,443 (82.6%) | 815 (74.1%) | 52 (81.3%) | 23 (74.2%) |
|  | Uninsured | 226 (3.3%) | 27 (4.2%) | 72 (3.5%) | 127 (3.1%) | 121 (3.6%) | 105 (3.0%) | 31 (16.1%) | 164 (3.0%) | 20 (1.8%) | <11 | <11 |
|  |  |  |  |  |  |  |  |  |  |  |  |  |
| California | Total | 75,247 | 9,239 | 24,944 | 41,064 | 33,355 | 41,892 | 21,345 | 35,801 | 5,214 | 10,357 | 318 |
|  | Medicaid | 15,781 (22.0%) | 2,389 (26.9%) | 5,352 (22.3%) | 8,040 (20.7%) | 6,856 (21.9%) | 8,925 (22.1%) | 7,356 (35.5%) | 4,842 (14.0%) | 1,437 (28.2%) | 1,828 (18.0%) | 73 (23.5%) |
|  | Other Insured | 55,106 (76.7%) | 6,396 (71.9%) | 18,326 (76.3%) | 30,384 (78.1%) | 24,072 (76.7%) | 31,034 (76.7%) | 12,950 (62.6%) | 29,350 (85.0%) | 3,601 (70.6%) | 8,247 (81.0%) | 234 (75.5%) |
|  | Uninsured | 929 (1.3%) | 109 (1.2%) | 344 (1.4%) | 476 (1.2%) | 448 (1.4%) | 481 (1.2%) | 386 (1.9%) | 346 (1.0%) | 60 (1.2%) | 107 (1.1%) | <11 |
|  |  |  |  |  |  |  |  |  |  |  |  |  |
| Colorado | Total | 10,764 | 1,309 | 3,561 | 5,894 | 4,935 | 5,829 | 1,634 | 8,185 | 462 | 245 | 76 |
|  | Medicaid | 1,908 (18.3%) | 288 (22.6%) | 613 (17.8%) | 1,007 (17.7%) | 846 (17.9%) | 1,062 (18.7%) | 508 (32.1%) | 1,154 (14.5%) | 149 (33.0%) | 42 (17.8%) | 24 (33.8%) |
|  | Other Insured | 8,145 (78.3%) | 929 (72.8%) | 2,703 (78.5%) | 4,513 (79.4%) | 3,707 (78.4%) | 4,438 (78.2%) | 918 (58.0%) | 6,613 (83.4%) | 294 (65.0%) | 188 (79.7%) | 46 (64.8%) |
|  | Uninsured | 348 (3.3%) | 59 (4.6%) | 126 (3.7%) | 163 (2.9%) | 176 (3.7%) | 172 (3.0%) | 158 (10.0%) | 167 (2.1%) | <11 | <11 | <11 |
|  |  |  |  |  |  |  |  |  |  |  |  |  |
| Connecticut | Total | 8,993 | 805 | 2,846 | 5,342 | 4,219 | 4,774 | 1,060 | 6,626 | 924 | 219 | <11 |
|  | Medicaid | 1,700 (20.1%) | 207 (27.6%) | 564 (21.1%) | 929 (18.5%) | 778 (19.8%) | 922 (20.5%) | 402 (40.3%) | 940 (15.0%) | 293 (33.8%) | 49 (23.2%) | <11 |
|  | Other Insured | 6,526 (77.3%) | 502 (66.8%) | 2,046 (76.5%) | 3,978 (79.3%) | 3,046 (77.4%) | 3,480 (77.2%) | 518 (51.9%) | 5,233 (83.6%) | 531 (61.3%) | 156 (73.9%) | <11 |
|  | Uninsured | 215 (2.5%) | 42 (5.6%) | 66 (2.5%) | 107 (2.1%) | 109 (2.8%) | 106 (2.4%) | 78 (7.8%) | 83 (1.3%) | 42 (4.8%) | <11 | <11 |
|  |  |  |  |  |  |  |  |  |  |  |  |  |
| Delaware | Total | 2,434 | 219 | 732 | 1,483 | 1,159 | 1,275 | 117 | 1,681 | 486 | 51 | <11 |
|  | Medicaid | 201 (9.5%) | 23 (11.9%) | 60 (9.2%) | 118 (9.3%) | 102 (10.8%) | 99 (8.5%) | 22 (21.6%) | 130 (8.6%) | 47 (10.7%) | <11 | <11 |
|  | Other Insured | 1,873 (88.5%) | 164 (85.0%) | 574 (88.0%) | 1,135 (89.2%) | 829 (87.4%) | 1,044 (89.3%) | 72 (70.6%) | 1,351 (89.8%) | 385 (87.5%) | 46 (93.9%) | <11 |
|  | Uninsured | 43 (2.0%) | <11 | 18 (2.8%) | 19 (1.5%) | 17 (1.8%) | 26 (2.2%) | <11 | 24 (1.6%) | <11 | <11 | <11 |
|  |  |  |  |  |  |  |  |  |  |  |  |  |
| District of Columbia | Total | 1,331 | 200 | 406 | 725 | 606 | 725 | 81 | 411 | 764 | 33 | <11 |
|  | Medicaid | 466 (37.9%) | 56 (29.9%) | 128 (33.9%) | 282 (42.3%) | 204 (37.0%) | 262 (38.5%) | 40 (50.6%) | 21 (5.6%) | 394 (53.5%) | <11 | <11 |
|  | Other Insured | 753 (61.2%) | 127 (67.9%) | 247 (65.3%) | 379 (56.9%) | 339 (61.5%) | 414 (60.9%) | 38 (48.1%) | 348 (93.3%) | 336 (45.7%) | 22 (75.9%) | <11 |
|  | Uninsured | 12 (1.0%) | <11 | <11 | <11 | <11 | <11 | <11 | <11 | <11 | <11 | <11 |
|  |  |  |  |  |  |  |  |  |  |  |  |  |
| Florida | Total | 49,231 | 4,686 | 15,459 | 29,086 | 22,416 | 26,815 | 9,772 | 30,755 | 6,545 | 890 | 85 |
|  | Medicaid | 5,278 (12.1%) | 604 (14.9%) | 1,715 (12.5%) | 2,959 (11.5%) | 2,462 (12.5%) | 2,816 (11.8%) | 1,173 (13.0%) | 2,866 (10.6%) | 1,131 (18.8%) | 42 (5.0%) | 11 (13.4%) |
|  | Other Insured | 35,498 (81.5%) | 3,100 (76.6%) | 11,041 (80.4%) | 21,357 (82.9%) | 15,829 (80.3%) | 19,669 (82.5%) | 7,200 (79.6%) | 22,577 (83.5%) | 4,436 (73.9%) | 769 (91.0%) | 67 (81.7%) |
|  | Uninsured | 2,788 (6.4%) | 344 (8.5%) | 983 (7.2%) | 1,461 (5.7%) | 1,422 (7.2%) | 1,366 (5.7%) | 674 (7.4%) | 1,599 (5.9%) | 435 (7.2%) | 34 (4.0%) | <11 |
|  |  |  |  |  |  |  |  |  |  |  |  |  |
| Georgia | Total | 24,592 | 2,522 | 8,117 | 13,953 | 11,871 | 12,721 | 1,302 | 14,665 | 7,774 | 702 | 25 |
|  | Medicaid | 2,945 (13.6%) | 352 (15.5%) | 863 (12.0%) | 1,730 (14.2%) | 1,253 (12.4%) | 1,692 (14.6%) | 142 (11.9%) | 1,324 (10.4%) | 1,399 (19.9%) | 70 (10.9%) | <11 |
|  | Other Insured | 16,601 (76.6%) | 1,627 (71.7%) | 5,596 (77.8%) | 9,378 (76.8%) | 7,713 (76.3%) | 8,888 (76.8%) | 693 (58.1%) | 10,375 (81.6%) | 4,950 (70.2%) | 512 (79.4%) | 16 (69.6%) |
|  | Uninsured | 2,137 (9.9%) | 291 (12.8%) | 737 (10.2%) | 1,109 (9.1%) | 1,143 (11.3%) | 994 (8.6%) | 358 (30.0%) | 1,009 (7.9%) | 698 (9.9%) | 63 (9.8%) | <11 |
|  |  |  |  |  |  |  |  |  |  |  |  |  |
| Hawaii | Total | 3,050 | 347 | 1,009 | 1,694 | 1,321 | 1,729 | 202 | 812 | 42 | 1,892 | 13 |
|  | Medicaid | 549 (20.5%) | 77 (24.8%) | 180 (19.9%) | 292 (20.0%) | 227 (20.6%) | 322 (20.5%) | 54 (29.5%) | 127 (18.4%) | 11 (28.9%) | 347 (20.0%) | <11 |
|  | Other Insured | 2,101 (78.6%) | 230 (74.2%) | 713 (78.9%) | 1,158 (79.3%) | 862 (78.2%) | 1,239 (78.8%) | 127 (69.4%) | 556 (80.6%) | 25 (65.8%) | 1,379 (79.3%) | <11 |
|  | Uninsured | 24 (0.9%) | <11 | 11 (1.2%) | <11 | 13 (1.2%) | 11 (0.7%) | <11 | <11 | <11 | 12 (0.7%) | <11 |
|  |  |  |  |  |  |  |  |  |  |  |  |  |
| Idaho | Total | 3,522 | 417 | 1,048 | 2,057 | 1,694 | 1,828 | 245 | 3,137 | 19 | 39 | 25 |
|  | Medicaid | 427 (13.4%) | 60 (16.2%) | 134 (13.9%) | 233 (12.6%) | 186 (12.5%) | 241 (14.2%) | 47 (20.3%) | 357 (12.5%) | <11 | <11 | <11 |
|  | Other Insured | 2,528 (79.4%) | 269 (72.7%) | 762 (79.0%) | 1,497 (81.0%) | 1,194 (80.1%) | 1,334 (78.8%) | 150 (64.7%) | 2,300 (80.8%) | 14 (82.4%) | 31 (79.5%) | 16 (64.0%) |
|  | Uninsured | 228 (7.2%) | 41 (11.1%) | 69 (7.2%) | 118 (6.4%) | 111 (7.4%) | 117 (6.9%) | 35 (15.1%) | 190 (6.7%) | <11 | <11 | <11 |
|  |  |  |  |  |  |  |  |  |  |  |  |  |
| Illinois | Total | 29,628 | 3,017 | 9,348 | 17,263 | 13,844 | 15,784 | 3,187 | 20,411 | 4,497 | 1,039 | 45 |
|  | Medicaid | 4,873 (17.2%) | 541 (19.1%) | 1,430 (16.0%) | 2,902 (17.6%) | 2,344 (17.9%) | 2,529 (16.6%) | 607 (19.7%) | 2,549 (13.1%) | 1,516 (34.7%) | 147 (14.6%) | 12 (28.6%) |
|  | Other Insured | 22,316 (78.9%) | 2,172 (76.5%) | 7,069 (79.1%) | 13,075 (79.2%) | 10,194 (77.9%) | 12,122 (79.8%) | 2,031 (65.9%) | 16,537 (84.9%) | 2,700 (61.8%) | 775 (76.8%) | 28 (66.7%) |
|  | Uninsured | 1,094 (3.9%) | 126 (4.4%) | 434 (4.9%) | 534 (3.2%) | 548 (4.2%) | 546 (3.6%) | 446 (14.5%) | 393 (2.0%) | 151 (3.5%) | 87 (8.6%) | <11 |
|  |  |  |  |  |  |  |  |  |  |  |  |  |
| Indiana | Total | 14,226 | 1,432 | 4,374 | 8,420 | 6,593 | 7,633 | 536 | 12,156 | 1,197 | 174 | 11 |
|  | Medicaid | 2,331 (16.9%) | 282 (20.2%) | 688 (16.2%) | 1,361 (16.6%) | 1,021 (16.0%) | 1,310 (17.6%) | 142 (28.0%) | 1,792 (15.1%) | 344 (29.8%) | 26 (15.1%) | <11 |
|  | Other Insured | 10,996 (79.5%) | 1,049 (75.0%) | 3,371 (79.3%) | 6,576 (80.4%) | 5,108 (80.0%) | 5,888 (79.1%) | 321 (63.2%) | 9,653 (81.5%) | 771 (66.9%) | 139 (80.8%) | <11 |
|  | Uninsured | 503 (3.6%) | 68 (4.9%) | 190 (4.5%) | 245 (3.0%) | 257 (4.0%) | 246 (3.3%) | 45 (8.9%) | 406 (3.4%) | 38 (3.3%) | <11 | <11 |
|  |  |  |  |  |  |  |  |  |  |  |  |  |
| Iowa | Total | 7,680 | 815 | 2,302 | 4,563 | 3,697 | 3,983 | 180 | 7,065 | 253 | 96 | 14 |
|  | Medicaid | 1,149 (15.9%) | 160 (21.5%) | 353 (16.4%) | 636 (14.7%) | 548 (15.8%) | 601 (16.0%) | 55 (32.5%) | 962 (14.4%) | 101 (41.6%) | 18 (19.6%) | <11 |
|  | Other Insured | 5,961 (82.6%) | 567 (76.1%) | 1,772 (82.2%) | 3,622 (83.9%) | 2,865 (82.7%) | 3,096 (82.5%) | 99 (58.6%) | 5,631 (84.4%) | 137 (56.4%) | 71 (77.2%) | <11 |
|  | Uninsured | 107 (1.5%) | 18 (2.4%) | 31 (1.4%) | 58 (1.3%) | 50 (1.4%) | 57 (1.5%) | 15 (8.9%) | 82 (1.2%) | <11 | <11 | <11 |
|  |  |  |  |  |  |  |  |  |  |  |  |  |
| Kentucky | Total | 12,346 | 1,149 | 3,946 | 7,251 | 5,939 | 6,407 | 180 | 10,957 | 945 | 83 | <11 |
|  | Medicaid | 3,058 (26.0%) | 336 (30.7%) | 1,059 (28.0%) | 1,663 (24.2%) | 1,363 (24.4%) | 1,695 (27.4%) | 51 (29.5%) | 2,676 (25.4%) | 298 (32.4%) | 22 (26.5%) | <11 |
|  | Other Insured | 8,502 (72.3%) | 738 (67.4%) | 2,652 (70.1%) | 5,112 (74.2%) | 4,124 (73.8%) | 4,378 (70.8%) | 88 (50.9%) | 7,696 (73.1%) | 603 (65.6%) | 59 (71.1%) | <11 |
|  | Uninsured | 206 (1.8%) | 21 (1.9%) | 74 (2.0%) | 111 (1.6%) | 99 (1.8%) | 107 (1.7%) | 34 (19.7%) | 150 (1.4%) | 18 (2.0%) | <11 | <11 |
|  |  |  |  |  |  |  |  |  |  |  |  |  |
| Louisiana | Total | 12,099 | 1,208 | 3,664 | 7,227 | 6,195 | 5,904 | 322 | 7,650 | 3,858 | 139 | 22 |
|  | Medicaid | 3,215 (27.6%) | 374 (32.4%) | 977 (27.7%) | 1,864 (26.7%) | 1,516 (25.5%) | 1,699 (29.7%) | 76 (25.0%) | 1,527 (20.8%) | 1,556 (41.2%) | 41 (30.4%) | <11 |
|  | Other Insured | 8,005 (68.7%) | 714 (61.9%) | 2,409 (68.2%) | 4,882 (70.0%) | 4,194 (70.6%) | 3,811 (66.7%) | 162 (53.3%) | 5,608 (76.3%) | 2,085 (55.2%) | 80 (59.3%) | 18 (81.8%) |
|  | Uninsured | 436 (3.7%) | 66 (5.7%) | 144 (4.1%) | 226 (3.2%) | 230 (3.9%) | 206 (3.6%) | 66 (21.7%) | 215 (2.9%) | 135 (3.6%) | 14 (10.4%) | <11 |
|  |  |  |  |  |  |  |  |  |  |  |  |  |
| Maine | Total | 3,697 | 309 | 1,059 | 2,329 | 1,731 | 1,966 | 25 | 3,551 | 36 | 22 | 23 |
|  | Medicaid | 791 (22.3%) | 70 (23.9%) | 235 (23.0%) | 486 (21.7%) | 374 (22.3%) | 417 (22.2%) | <11 | 756 (22.0%) | 13 (38.2%) | <11 | 12 (57.1%) |
|  | Other Insured | 2,609 (73.4%) | 207 (70.6%) | 744 (72.7%) | 1,658 (74.1%) | 1,229 (73.4%) | 1,380 (73.4%) | 20 (83.3%) | 2,539 (73.9%) | 14 (41.2%) | 14 (63.6%) | <11 |
|  | Uninsured | 154 (4.3%) | 16 (5.5%) | 44 (4.3%) | 94 (4.2%) | 71 (4.2%) | 83 (4.4%) | <11 | 141 (4.1%) | <11 | <11 | <11 |
|  |  |  |  |  |  |  |  |  |  |  |  |  |
| Maryland | Total | 14,689 | 1,414 | 4,659 | 8,616 | 6,807 | 7,882 | 747 | 8,496 | 4,620 | 690 | 12 |
|  | Medicaid | 1,732 (12.9%) | 210 (16.2%) | 530 (12.3%) | 992 (12.6%) | 794 (12.9%) | 938 (12.8%) | 114 (16.7%) | 737 (9.5%) | 819 (19.0%) | 56 (8.6%) | <11 |
|  | Other Insured | 11,510 (85.5%) | 1,044 (80.4%) | 3,694 (85.9%) | 6,772 (86.0%) | 5,232 (85.1%) | 6,278 (85.8%) | 498 (72.9%) | 6,925 (89.5%) | 3,440 (79.6%) | 586 (89.6%) | <11 |
|  | Uninsured | 227 (1.7%) | 44 (3.4%) | 74 (1.7%) | 109 (1.4%) | 123 (2.0%) | 104 (1.4%) | 71 (10.4%) | 77 (1.0%) | 62 (1.4%) | 12 (1.8%) | <11 |
|  |  |  |  |  |  |  |  |  |  |  |  |  |
| Massachusetts | Total | 15,500 | 1,470 | 4,959 | 9,071 | 7,080 | 8,420 | 1,105 | 12,243 | 1,084 | 622 | 20 |
|  | Medicaid | 2,854 (19.0%) | 286 (19.8%) | 872 (18.0%) | 1,696 (19.4%) | 1,341 (19.7%) | 1,513 (18.4%) | 468 (43.6%) | 1,902 (16.0%) | 282 (27.3%) | 96 (15.7%) | <11 |
|  | Other Insured | 12,089 (80.5%) | 1,141 (79.2%) | 3,942 (81.5%) | 7,006 (80.2%) | 5,421 (79.7%) | 6,668 (81.2%) | 595 (55.5%) | 9,929 (83.6%) | 738 (71.5%) | 512 (83.8%) | 15 (75.0%) |
|  | Uninsured | 70 (0.5%) | 14 (1.0%) | 21 (0.4%) | 35 (0.4%) | 38 (0.6%) | 32 (0.4%) | <11 | 40 (0.3%) | 12 (1.2%) | <11 | <11 |
|  |  |  |  |  |  |  |  |  |  |  |  |  |
| Michigan | Total | 23,171 | 2,060 | 7,185 | 13,926 | 11,204 | 11,967 | 502 | 18,409 | 3,082 | 387 | 107 |
|  | Medicaid | 3,619 (19.9%) | 406 (25.0%) | 1,175 (20.9%) | 2,038 (18.6%) | 1,713 (19.5%) | 1,906 (20.3%) | 136 (34.9%) | 2,700 (17.8%) | 652 (33.4%) | 51 (17.8%) | 25 (27.2%) |
|  | Other Insured | 14,310 (78.7%) | 1,193 (73.4%) | 4,369 (77.6%) | 8,748 (80.1%) | 6,926 (78.9%) | 7,384 (78.5%) | 244 (62.6%) | 12,236 (80.9%) | 1,266 (64.8%) | 232 (81.1%) | 66 (71.7%) |
|  | Uninsured | 254 (1.4%) | 27 (1.7%) | 85 (1.5%) | 142 (1.3%) | 134 (1.5%) | 120 (1.3%) | <11 | 197 (1.3%) | 35 (1.8%) | <11 | <11 |
|  |  |  |  |  |  |  |  |  |  |  |  |  |
| Mississippi | Total | 7,413 | 629 | 2,266 | 4,518 | 3,712 | 3,701 | 83 | 4,400 | 2,838 | 61 | 18 |
|  | Medicaid | 1,464 (20.1%) | 140 (22.7%) | 410 (18.4%) | 914 (20.6%) | 743 (20.5%) | 721 (19.8%) | 11 (13.4%) | 621 (14.4%) | 819 (29.2%) | <11 | <11 |
|  | Other Insured | 5,262 (72.3%) | 422 (68.3%) | 1,625 (72.9%) | 3,215 (72.6%) | 2,595 (71.5%) | 2,667 (73.2%) | 47 (57.3%) | 3,379 (78.6%) | 1,767 (63.0%) | 45 (73.8%) | 16 (88.9%) |
|  | Uninsured | 549 (7.5%) | 56 (9.1%) | 193 (8.7%) | 300 (6.8%) | 293 (8.1%) | 256 (7.0%) | 24 (29.3%) | 300 (7.0%) | 217 (7.7%) | <11 | <11 |
|  |  |  |  |  |  |  |  |  |  |  |  |  |
| Missouri | Total | 14,499 | 1,411 | 4,497 | 8,591 | 6,702 | 7,797 | 266 | 12,116 | 1,792 | 170 | 17 |
|  | Medicaid | 2,274 (16.3%) | 232 (16.9%) | 683 (15.7%) | 1,359 (16.5%) | 1,058 (16.5%) | 1,216 (16.1%) | 47 (18.1%) | 1,663 (14.2%) | 522 (29.8%) | 23 (13.9%) | <11 |
|  | Other Insured | 10,908 (78.0%) | 1,018 (74.0%) | 3,431 (78.6%) | 6,459 (78.4%) | 4,956 (77.3%) | 5,952 (78.7%) | 143 (55.0%) | 9,455 (81.0%) | 1,076 (61.5%) | 131 (79.4%) | <11 |
|  | Uninsured | 794 (5.7%) | 125 (9.1%) | 250 (5.7%) | 419 (5.1%) | 400 (6.2%) | 394 (5.2%) | 70 (26.9%) | 559 (4.8%) | 151 (8.6%) | 11 (6.7%) | <11 |
|  |  |  |  |  |  |  |  |  |  |  |  |  |
| Montana | Total | 2,455 | 264 | 686 | 1,505 | 1,176 | 1,279 | 32 | 2,202 | 14 | 13 | 119 |
|  | Medicaid | 514 (21.9%) | 64 (25.4%) | 160 (24.2%) | 290 (20.2%) | 235 (21.1%) | 279 (22.6%) | <11 | 431 (20.3%) | <11 | <11 | 54 (48.6%) |
|  | Other Insured | 1,749 (74.5%) | 176 (69.8%) | 479 (72.5%) | 1,094 (76.3%) | 833 (74.8%) | 916 (74.2%) | 22 (68.8%) | 1,620 (76.2%) | <11 | <11 | 55 (49.5%) |
|  | Uninsured | 84 (3.6%) | 12 (4.8%) | 22 (3.3%) | 50 (3.5%) | 45 (4.0%) | 39 (3.2%) | <11 | 76 (3.6%) | <11 | <11 | <11 |
|  |  |  |  |  |  |  |  |  |  |  |  |  |
| Nebraska | Total | 4,300 | 530 | 1,251 | 2,519 | 2,042 | 2,258 | 248 | 3,654 | 175 | 62 | 33 |
|  | Medicaid | 288 (7.9%) | 41 (9.4%) | 89 (8.2%) | 158 (7.3%) | 133 (7.9%) | 155 (7.8%) | 25 (12.1%) | 220 (7.0%) | 18 (10.8%) | <11 | 12 (41.4%) |
|  | Other Insured | 3,176 (86.6%) | 361 (83.0%) | 923 (85.5%) | 1,892 (88.0%) | 1,450 (86.0%) | 1,726 (87.2%) | 138 (67.0%) | 2,809 (88.8%) | 133 (79.6%) | 47 (82.5%) | 16 (55.2%) |
|  | Uninsured | 202 (5.5%) | 33 (7.6%) | 68 (6.3%) | 101 (4.7%) | 104 (6.2%) | 98 (5.0%) | 43 (20.9%) | 134 (4.2%) | 16 (9.6%) | <11 | <11 |
|  |  |  |  |  |  |  |  |  |  |  |  |  |
| New Hampshire | Total | 3,626 | 309 | 1,045 | 2,272 | 1,727 | 1,899 | 81 | 3,390 | 32 | 39 | <11 |
|  | Medicaid | 418 (12.2%) | 46 (15.4%) | 111 (11.2%) | 261 (12.2%) | 182 (11.2%) | 236 (13.0%) | 17 (21.8%) | 384 (11.8%) | <11 | <11 | <11 |
|  | Other Insured | 2,926 (85.2%) | 247 (82.9%) | 852 (85.6%) | 1,827 (85.3%) | 1,378 (85.2%) | 1,548 (85.2%) | 57 (73.1%) | 2,778 (85.6%) | 22 (75.9%) | 31 (81.6%) | <11 |
|  | Uninsured | 91 (2.6%) | <11 | 32 (3.2%) | 54 (2.5%) | 58 (3.6%) | 33 (1.8%) | <11 | 85 (2.6%) | <11 | <11 | <11 |
|  |  |  |  |  |  |  |  |  |  |  |  |  |
| New Jersey | Total | 22,734 | 2,245 | 7,412 | 13,077 | 10,814 | 11,920 | 3,206 | 14,335 | 2,963 | 1,412 | 25 |
|  | Medicaid | 2,452 (12.1%) | 322 (15.7%) | 813 (12.1%) | 1,317 (11.5%) | 1,137 (12.4%) | 1,315 (11.9%) | 605 (20.7%) | 1,070 (8.2%) | 614 (23.0%) | 132 (10.1%) | <11 |
|  | Other Insured | 16,999 (83.8%) | 1,589 (77.4%) | 5,619 (83.4%) | 9,791 (85.2%) | 7,702 (83.7%) | 9,297 (83.9%) | 1,948 (66.6%) | 11,790 (89.9%) | 1,931 (72.3%) | 1,110 (84.7%) | 15 (68.2%) |
|  | Uninsured | 836 (4.1%) | 143 (7.0%) | 304 (4.5%) | 389 (3.4%) | 362 (3.9%) | 474 (4.3%) | 373 (12.7%) | 255 (1.9%) | 126 (4.7%) | 69 (5.3%) | <11 |
|  |  |  |  |  |  |  |  |  |  |  |  |  |
| New Mexico | Total | 4,046 | 457 | 1,284 | 2,305 | 1,797 | 2,249 | 1,706 | 1,844 | 69 | 69 | 226 |
|  | Medicaid | 875 (25.5%) | 145 (36.7%) | 292 (26.7%) | 438 (22.5%) | 390 (26.5%) | 485 (24.7%) | 411 (27.3%) | 325 (20.7%) | 22 (37.9%) | 15 (25.9%) | 91 (43.8%) |
|  | Other Insured | 2,423 (70.5%) | 225 (57.0%) | 740 (67.6%) | 1,458 (74.9%) | 1,017 (69.0%) | 1,406 (71.7%) | 995 (66.1%) | 1,210 (77.2%) | 36 (62.1%) | 40 (69.0%) | 114 (54.8%) |
|  | Uninsured | 138 (4.0%) | 25 (6.3%) | 62 (5.7%) | 51 (2.6%) | 67 (4.5%) | 71 (3.6%) | 99 (6.6%) | 32 (2.0%) | <11 | <11 | <11 |
|  |  |  |  |  |  |  |  |  |  |  |  |  |
| New York | Total | 49,938 | 5,566 | 15,898 | 28,474 | 23,215 | 26,723 | 6,803 | 30,833 | 7,552 | 3,715 | 99 |
|  | Medicaid | 11,424 (24.1%) | 1,540 (29.2%) | 3,755 (24.8%) | 6,129 (22.7%) | 5,115 (23.5%) | 6,309 (24.6%) | 2,685 (41.3%) | 4,393 (14.9%) | 2,663 (37.1%) | 1,433 (40.4%) | 33 (35.1%) |
|  | Other Insured | 35,396 (74.7%) | 3,647 (69.1%) | 11,168 (73.9%) | 20,581 (76.2%) | 16,383 (75.2%) | 19,013 (74.3%) | 3,650 (56.1%) | 24,834 (84.3%) | 4,425 (61.6%) | 2,079 (58.6%) | 61 (64.9%) |
|  | Uninsured | 569 (1.2%) | 91 (1.7%) | 196 (1.3%) | 282 (1.0%) | 293 (1.3%) | 276 (1.1%) | 171 (2.6%) | 245 (0.8%) | 99 (1.4%) | 36 (1.0%) | <11 |
|  |  |  |  |  |  |  |  |  |  |  |  |  |
| North Carolina | Total | 25,190 | 2,288 | 8,087 | 14,815 | 12,032 | 13,158 | 1,105 | 17,443 | 5,487 | 399 | 227 |
|  | Medicaid | 3,479 (14.3%) | 362 (16.4%) | 1,113 (14.2%) | 2,004 (14.0%) | 1,701 (14.7%) | 1,778 (13.9%) | 168 (15.6%) | 1,962 (11.5%) | 1,232 (22.9%) | 40 (10.1%) | 52 (23.7%) |
|  | Other Insured | 19,394 (79.5%) | 1,628 (73.7%) | 6,176 (78.6%) | 11,590 (81.0%) | 9,078 (78.5%) | 10,316 (80.5%) | 598 (55.5%) | 14,302 (83.8%) | 3,809 (70.9%) | 329 (83.1%) | 152 (69.4%) |
|  | Uninsured | 1,508 (6.2%) | 218 (9.9%) | 572 (7.3%) | 718 (5.0%) | 784 (6.8%) | 724 (5.6%) | 312 (28.9%) | 803 (4.7%) | 333 (6.2%) | 27 (6.8%) | 15 (6.8%) |
|  |  |  |  |  |  |  |  |  |  |  |  |  |
| North Dakota | Total | 1,565 | 175 | 430 | 960 | 756 | 809 | 26 | 1,379 | 28 | 11 | 75 |
|  | Medicaid | 145 (9.5%) | 15 (8.8%) | 47 (11.3%) | 83 (8.9%) | 66 (8.9%) | 79 (10.1%) | <11 | 112 (8.4%) | <11 | <11 | 11 (15.1%) |
|  | Other Insured | 1,323 (87.0%) | 145 (84.8%) | 356 (85.6%) | 822 (88.1%) | 641 (86.9%) | 682 (87.2%) | 14 (56.0%) | 1,190 (88.9%) | 12 (44.4%) | <11 | 59 (80.8%) |
|  | Uninsured | 52 (3.4%) | 11 (6.4%) | 13 (3.1%) | 28 (3.0%) | 31 (4.2%) | 21 (2.7%) | <11 | 37 (2.8%) | <11 | <11 | <11 |
|  |  |  |  |  |  |  |  |  |  |  |  |  |
| Ohio | Total | 29,523 | 2,865 | 8,936 | 17,722 | 14,024 | 15,499 | 449 | 24,718 | 3,420 | 343 | 30 |
|  | Medicaid | 5,663 (19.7%) | 721 (25.8%) | 1,763 (20.2%) | 3,179 (18.5%) | 2,549 (18.8%) | 3,114 (20.5%) | 132 (30.6%) | 4,145 (17.1%) | 1,248 (37.3%) | 72 (21.1%) | <11 |
|  | Other Insured | 22,298 (77.7%) | 1,989 (71.1%) | 6,740 (77.2%) | 13,569 (79.0%) | 10,608 (78.4%) | 11,690 (77.1%) | 246 (57.1%) | 19,480 (80.5%) | 2,022 (60.5%) | 251 (73.6%) | 23 (76.7%) |
|  | Uninsured | 736 (2.6%) | 87 (3.1%) | 229 (2.6%) | 420 (2.4%) | 376 (2.8%) | 360 (2.4%) | 53 (12.3%) | 574 (2.4%) | 72 (2.2%) | 18 (5.3%) | <11 |
|  |  |  |  |  |  |  |  |  |  |  |  |  |
| Oklahoma | Total | 8,722 | 888 | 2,691 | 5,143 | 4,158 | 4,564 | 404 | 6,370 | 654 | 131 | 692 |
|  | Medicaid | 922 (11.2%) | 108 (12.9%) | 304 (11.9%) | 510 (10.6%) | 371 (9.6%) | 551 (12.7%) | 59 (15.7%) | 610 (10.1%) | 108 (17.5%) | 15 (12.3%) | 86 (12.9%) |
|  | Other Insured | 6,733 (81.9%) | 657 (78.7%) | 2,052 (80.1%) | 4,024 (83.5%) | 3,186 (82.4%) | 3,547 (81.6%) | 250 (66.5%) | 5,041 (83.2%) | 456 (73.9%) | 102 (83.6%) | 562 (84.5%) |
|  | Uninsured | 561 (6.8%) | 70 (8.4%) | 206 (8.0%) | 285 (5.9%) | 311 (8.0%) | 250 (5.7%) | 67 (17.8%) | 406 (6.7%) | 53 (8.6%) | <11 | 17 (2.6%) |
|  |  |  |  |  |  |  |  |  |  |  |  |  |
| Oregon | Total | 8,605 | 988 | 2,693 | 4,924 | 3,856 | 4,749 | 647 | 7,219 | 174 | 338 | 133 |
|  | Medicaid | 1,644 (19.9%) | 223 (23.4%) | 569 (22.0%) | 852 (18.1%) | 781 (21.2%) | 863 (18.9%) | 209 (33.5%) | 1,251 (18.1%) | 66 (39.8%) | 61 (18.3%) | 39 (30.0%) |
|  | Other Insured | 6,439 (78.1%) | 718 (75.2%) | 1,952 (75.4%) | 3,769 (80.2%) | 2,824 (76.8%) | 3,615 (79.2%) | 386 (62.0%) | 5,566 (80.3%) | 96 (57.8%) | 260 (78.1%) | 89 (68.5%) |
|  | Uninsured | 159 (1.9%) | 14 (1.5%) | 67 (2.6%) | 78 (1.7%) | 71 (1.9%) | 88 (1.9%) | 28 (4.5%) | 112 (1.6%) | <11 | 12 (3.6%) | <11 |
|  |  |  |  |  |  |  |  |  |  |  |  |  |
| Pennsylvania | Total | 31,662 | 2,974 | 9,524 | 19,164 | 15,056 | 16,606 | 1,368 | 25,768 | 3,253 | 598 | 32 |
|  | Medicaid | 4,627 (15.2%) | 594 (20.6%) | 1,518 (16.5%) | 2,515 (13.7%) | 2,059 (14.4%) | 2,568 (16.0%) | 482 (36.8%) | 3,023 (12.1%) | 947 (30.5%) | 117 (19.9%) | 14 (45.2%) |
|  | Other Insured | 25,323 (83.3%) | 2,243 (77.7%) | 7,533 (82.0%) | 15,547 (84.9%) | 12,009 (84.0%) | 13,314 (82.7%) | 766 (58.5%) | 21,702 (86.7%) | 2,107 (67.8%) | 459 (77.9%) | 17 (54.8%) |
|  | Uninsured | 439 (1.4%) | 51 (1.8%) | 141 (1.5%) | 247 (1.3%) | 227 (1.6%) | 212 (1.3%) | 62 (4.7%) | 295 (1.2%) | 55 (1.8%) | 13 (2.2%) | <11 |
|  |  |  |  |  |  |  |  |  |  |  |  |  |
| Rhode Island | Total | 2,662 | 246 | 832 | 1,584 | 1,252 | 1,410 | 213 | 2,173 | 132 | 46 | <11 |
|  | Medicaid | 582 (22.2%) | 65 (26.6%) | 204 (24.9%) | 313 (20.1%) | 252 (20.5%) | 330 (23.7%) | 91 (42.7%) | 408 (19.1%) | 53 (41.1%) | 14 (30.4%) | <11 |
|  | Other Insured | 1,993 (76.1%) | 174 (71.3%) | 590 (72.1%) | 1,229 (78.9%) | 953 (77.7%) | 1,040 (74.7%) | 102 (47.9%) | 1,713 (80.0%) | 72 (55.8%) | 31 (67.4%) | <11 |
|  | Uninsured | 45 (1.7%) | <11 | 24 (2.9%) | 16 (1.0%) | 22 (1.8%) | 23 (1.7%) | 20 (9.4%) | 19 (0.9%) | <11 | <11 | <11 |
|  |  |  |  |  |  |  |  |  |  |  |  |  |
| South California | Total | 11,475 | 1,064 | 3,692 | 6,719 | 5,463 | 6,012 | 326 | 7,845 | 2,934 | 125 | 36 |
|  | Medicaid | 1,287 (12.4%) | 141 (14.8%) | 437 (12.9%) | 709 (11.7%) | 572 (11.8%) | 715 (12.9%) | 47 (15.9%) | 693 (9.6%) | 527 (19.7%) | <11 | <11 |
|  | Other Insured | 8,262 (79.7%) | 708 (74.4%) | 2,648 (78.3%) | 4,906 (81.3%) | 3,809 (78.8%) | 4,453 (80.5%) | 172 (58.3%) | 6,012 (83.7%) | 1,908 (71.2%) | 109 (87.2%) | 28 (84.8%) |
|  | Uninsured | 817 (7.9%) | 102 (10.7%) | 295 (8.7%) | 420 (7.0%) | 452 (9.4%) | 365 (6.6%) | 76 (25.8%) | 482 (6.7%) | 245 (9.1%) | <11 | <11 |
|  |  |  |  |  |  |  |  |  |  |  |  |  |
| South Dakota | Total | 1,822 | 203 | 538 | 1,081 | 875 | 947 | 36 | 1,611 | 19 | 14 | 133 |
|  | Medicaid | 135 (7.6%) | 24 (12.1%) | 34 (6.5%) | 77 (7.2%) | 52 (6.1%) | 83 (8.9%) | <11 | 87 (5.5%) | <11 | <11 | 36 (27.7%) |
|  | Other Insured | 1,575 (88.1%) | 162 (81.4%) | 461 (87.8%) | 952 (89.6%) | 759 (88.9%) | 816 (87.5%) | 20 (57.1%) | 1,434 (90.8%) | <11 | 11 (78.6%) | 91 (70.0%) |
|  | Uninsured | 77 (4.3%) | 13 (6.5%) | 30 (5.7%) | 34 (3.2%) | 43 (5.0%) | 34 (3.6%) | <11 | 59 (3.7%) | <11 | <11 | <11 |
|  |  |  |  |  |  |  |  |  |  |  |  |  |
| Tennessee | Total | 16,207 | 1,472 | 5,140 | 9,595 | 7,832 | 8,375 | 347 | 12,685 | 2,536 | 159 | 15 |
|  | Medicaid | 1,654 (11.2%) | 221 (16.6%) | 543 (11.6%) | 890 (10.2%) | 690 (9.8%) | 964 (12.5%) | 30 (9.5%) | 1,178 (10.0%) | 417 (17.5%) | 17 (11.6%) | <11 |
|  | Other Insured | 11,835 (80.4%) | 976 (73.4%) | 3,697 (79.1%) | 7,162 (82.1%) | 5,633 (80.4%) | 6,202 (80.4%) | 164 (51.7%) | 9,755 (82.8%) | 1,733 (72.7%) | 115 (78.8%) | <11 |
|  | Uninsured | 1,236 (8.4%) | 133 (10.0%) | 435 (9.3%) | 668 (7.7%) | 686 (9.8%) | 550 (7.1%) | 123 (38.8%) | 854 (7.2%) | 233 (9.8%) | 14 (9.6%) | <11 |
|  |  |  |  |  |  |  |  |  |  |  |  |  |
| Texas | Total | 55,565 | 6,804 | 18,772 | 29,989 | 25,780 | 29,785 | 15,470 | 29,481 | 7,642 | 1,958 | 215 |
|  | Medicaid | 4,605 (8.8%) | 682 (10.7%) | 1,499 (8.5%) | 2,424 (8.7%) | 1,951 (8.2%) | 2,654 (9.4%) | 1,746 (11.8%) | 1,705 (6.1%) | 1,055 (14.6%) | 74 (4.0%) | 14 (6.8%) |
|  | Other Insured | 40,549 (77.9%) | 4,592 (71.9%) | 13,644 (77.1%) | 22,313 (79.8%) | 18,537 (77.9%) | 22,012 (77.9%) | 9,620 (65.3%) | 23,790 (85.8%) | 5,139 (71.2%) | 1,575 (85.1%) | 167 (81.1%) |
|  | Uninsured | 6,890 (13.2%) | 1,111 (17.4%) | 2,557 (14.4%) | 3,222 (11.5%) | 3,294 (13.9%) | 3,596 (12.7%) | 3,374 (22.9%) | 2,245 (8.1%) | 1,026 (14.2%) | 201 (10.9%) | 25 (12.1%) |
|  |  |  |  |  |  |  |  |  |  |  |  |  |
| Utah | Total | 5,329 | 942 | 1,812 | 2,575 | 2,379 | 2,950 | 571 | 4,410 | 57 | 145 | 39 |
|  | Medicaid | 379 (8.6%) | 74 (9.5%) | 126 (8.3%) | 179 (8.5%) | 155 (8.3%) | 224 (8.9%) | 79 (16.1%) | 268 (7.3%) | <11 | 14 (10.6%) | 11 (30.6%) |
|  | Other Insured | 3,783 (86.0%) | 661 (85.2%) | 1,299 (85.3%) | 1,823 (86.8%) | 1,622 (86.7%) | 2,161 (85.4%) | 313 (63.9%) | 3,293 (89.4%) | 34 (79.1%) | 107 (81.1%) | 24 (66.7%) |
|  | Uninsured | 237 (5.4%) | 41 (5.3%) | 97 (6.4%) | 99 (4.7%) | 93 (5.0%) | 144 (5.7%) | 98 (20.0%) | 124 (3.4%) | <11 | 11 (8.3%) | <11 |
|  |  |  |  |  |  |  |  |  |  |  |  |  |
| Vermont | Total | 1,560 | 132 | 505 | 923 | 728 | 832 | 16 | 1,487 | 16 | 12 | <11 |
|  | Medicaid | 355 (23.3%) | 51 (38.9%) | 113 (23.0%) | 191 (21.2%) | 166 (23.4%) | 189 (23.1%) | <11 | 336 (23.0%) | <11 | <11 | <11 |
|  | Other Insured | 1,144 (75.0%) | 79 (60.3%) | 371 (75.4%) | 694 (76.9%) | 527 (74.3%) | 617 (75.5%) | <11 | 1,098 (75.3%) | <11 | <11 | <11 |
|  | Uninsured | 27 (1.8%) | <11 | <11 | 18 (2.0%) | 16 (2.3%) | 11 (1.3%) | <11 | 24 (1.6%) | <11 | <11 | <11 |
|  |  |  |  |  |  |  |  |  |  |  |  |  |
| Virginia | Total | 17,859 | 1,860 | 5,714 | 10,285 | 8,374 | 9,485 | 831 | 12,055 | 3,707 | 768 | 53 |
|  | Medicaid | 2,224 (13.2%) | 283 (16.1%) | 690 (12.7%) | 1,251 (12.9%) | 1,029 (13.2%) | 1,195 (13.1%) | 90 (11.3%) | 1,248 (10.9%) | 779 (21.9%) | 67 (9.3%) | <11 |
|  | Other Insured | 13,844 (81.9%) | 1,334 (75.9%) | 4,461 (82.4%) | 8,049 (82.8%) | 6,336 (81.5%) | 7,508 (82.3%) | 511 (64.1%) | 9,812 (85.7%) | 2,593 (72.9%) | 622 (86.4%) | 45 (84.9%) |
|  | Uninsured | 829 (4.9%) | 140 (8.0%) | 264 (4.9%) | 425 (4.4%) | 414 (5.3%) | 415 (4.6%) | 196 (24.6%) | 387 (3.4%) | 183 (5.1%) | 31 (4.3%) | <11 |
|  |  |  |  |  |  |  |  |  |  |  |  |  |
| Washington | Total | 15,660 | 1,916 | 4,911 | 8,833 | 7,021 | 8,639 | 1,032 | 11,978 | 694 | 1,314 | 280 |
|  | Medicaid | 2,738 (19.6%) | 407 (23.7%) | 859 (19.6%) | 1,472 (18.7%) | 1,219 (19.8%) | 1,519 (19.3%) | 327 (34.7%) | 1,847 (17.1%) | 203 (31.4%) | 236 (19.0%) | 98 (38.1%) |
|  | Other Insured | 10,958 (78.3%) | 1,269 (73.9%) | 3,431 (78.1%) | 6,258 (79.4%) | 4,786 (77.9%) | 6,172 (78.6%) | 534 (56.6%) | 8,761 (81.3%) | 427 (66.0%) | 978 (78.9%) | 152 (59.1%) |
|  | Uninsured | 300 (2.1%) | 42 (2.4%) | 103 (2.3%) | 155 (2.0%) | 137 (2.2%) | 163 (2.1%) | 82 (8.7%) | 163 (1.5%) | 17 (2.6%) | 26 (2.1%) | <11 |
|  |  |  |  |  |  |  |  |  |  |  |  |  |
| West Virginia | Total | 5,031 | 428 | 1,581 | 3,022 | 2,348 | 2,683 | 20 | 4,796 | 171 | 12 | <11 |
|  | Medicaid | 1,187 (24.2%) | 128 (30.7%) | 396 (25.8%) | 663 (22.5%) | 548 (23.9%) | 639 (24.5%) | <11 | 1,116 (23.8%) | 55 (32.5%) | <11 | <11 |
|  | Other Insured | 3,590 (73.2%) | 280 (67.1%) | 1,095 (71.2%) | 2,215 (75.1%) | 1,686 (73.6%) | 1,904 (72.9%) | 12 (63.2%) | 3,443 (73.6%) | 113 (66.9%) | <11 | <11 |
|  | Uninsured | 128 (2.6%) | <11 | 46 (3.0%) | 73 (2.5%) | 58 (2.5%) | 70 (2.7%) | <11 | 122 (2.6%) | <11 | <11 | <11 |
|  |  |  |  |  |  |  |  |  |  |  |  |  |
| Wisconsin | Total | 14,154 | 1,326 | 4,226 | 8,602 | 6,870 | 7,284 | 516 | 12,266 | 964 | 174 | 139 |
|  | Medicaid | 1,914 (13.7%) | 236 (18.2%) | 598 (14.3%) | 1,080 (12.8%) | 932 (13.8%) | 982 (13.7%) | 119 (23.6%) | 1,343 (11.1%) | 372 (39.0%) | 40 (23.0%) | 30 (22.1%) |
|  | Other Insured | 11,801 (84.7%) | 1,036 (79.8%) | 3,494 (83.7%) | 7,271 (86.0%) | 5,709 (84.6%) | 6,092 (84.8%) | 346 (68.5%) | 10,597 (87.7%) | 562 (58.9%) | 128 (73.6%) | 104 (76.5%) |
|  | Uninsured | 215 (1.5%) | 27 (2.1%) | 82 (2.0%) | 106 (1.3%) | 104 (1.5%) | 111 (1.5%) | 40 (7.9%) | 143 (1.2%) | 20 (2.1%) | <11 | <11 |
|  |  |  |  |  |  |  |  |  |  |  |  |  |
| Wyoming | Total | 1,250 | 137 | 363 | 750 | 597 | 653 | 65 | 1,146 | <11 | <11 | 13 |
|  | Medicaid | 80 (7.7%) | 14 (12.5%) | 32 (10.6%) | 34 (5.4%) | 25 (5.0%) | 55 (10.2%) | <11 | 69 (7.3%) | <11 | <11 | <11 |
|  | Other Insured | 870 (83.9%) | 93 (83.0%) | 233 (77.4%) | 544 (87.2%) | 416 (83.9%) | 454 (83.9%) | 43 (82.7%) | 800 (84.3%) | <11 | <11 | <11 |
|  | Uninsured | 87 (8.4%) | <11 | 36 (12.0%) | 46 (7.4%) | 55 (11.1%) | 32 (5.9%) | <11 | 80 (8.4%) | <11 | <11 | <11 |
|  |  |  |  |  |  |  |  |  |  |  |  |  |

Source: Authors’ analysis of Cancer Incidence in North America (CiNA) 2010-2019 compiled by the North American Association of Central Cancer Registries

Notes: Column percentage reported. Cases with unknown insurance status at diagnosis were excluded.

Figure S3. Trend of Percent Medicaid Insured and Uninsured By States Among Incident Cancer Cases Diagnosed at Ages 18-64 Years in 2010-2019, Including Cases With Unknown Insurance Status

1. Expansion States
2. Late Expansion States
3. Non-Expansion States


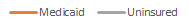


Source: Authors’ analysis of Cancer Incidence in North America (CiNA) 2010-2019 compiled by the North American Association of Central Cancer Registries

Notes: Cases with unknown insurance status at diagnosis were included.

Table S3. Insurance Coverage Among Incident Cancer Cases Diagnosed At Ages 18-64 Years by Age, Sex, Race/Ethnicity Across States in 2019, Including Cases With Unknown Insurance Status

|  |  | **Overall** | **18-39years** | **40-54years** | **55-64years** | **Male** | **Female** | **Hispanic** | **NH-White** | **NH-Black** | **NH-API** | **NH-AIAN** |
| --- | --- | --- | --- | --- | --- | --- | --- | --- | --- | --- | --- | --- |
| US | Total | 708,403 | 74,146 | 224,911 | 409,346 | 331,008 | 377,395 | 81,513 | 484,126 | 93,926 | 30,345 | 4,084 |
|  | Medicaid | 111,393 (15.7%) | 14,284 (19.3%) | 35,751 (15.9%) | 61,358 (15.0%) | 50,387 (15.2%) | 61,006 (16.2%) | 19,607 (24.1%) | 60,887 (12.6%) | 23,231 (24.7%) | 5,361 (17.7%) | 1,037 (25.4%) |
|  | Other Insured | 519,730 (73.4%) | 50,813 (68.5%) | 164,518 (73.1%) | 304,399 (74.4%) | 239,421 (72.3%) | 280,309 (74.3%) | 49,670 (60.9%) | 378,876 (78.3%) | 60,036 (63.9%) | 22,830 (75.2%) | 2,747 (67.3%) |
|  | Uninsured | 28,923 (4.1%) | 4,084 (5.5%) | 10,341 (4.6%) | 14,498 (3.5%) | 14,619 (4.4%) | 14,304 (3.8%) | 8,056 (9.9%) | 14,613 (3.0%) | 4,889 (5.2%) | 901  (3.0%) | 114 (2.8%) |
|  | Unknown | 48,357 (6.8%) | 4,954 (6.7%) | 14,301 (6.4%) | 29,091 (7.1%) | 26,581 (8.0%) | 21,776 (5.8%) | 4,154 (5.1%) | 29,750 (6.1%) | 5,705 (6.1%) | 1,156 (3.8%) | 100 (2.4%) |
| Alabama | Total | 11,913 | 1,092 | 3,791 | 7,030 | 5,821 | 6,092 | 179 | 8,254 | 3,076 | 113 | <11 |
|  | Medicaid | 1,428 (12.0%) | 167 (15.3%) | 458  (12.1%) | 803  (11.4%) | 612  (10.5%) | 816  (13.4%) | 21  (11.7%) | 794  (9.6%) | 598 (19.4%) | 11  (9.7%) | <11 |
|  | Other Insured | 8,587 (72.1%) | 704 (64.5%) | 2,714 (71.6%) | 5,169 (73.5%) | 4,145 (71.2%) | 4,442 (72.9%) | 90  (50.3%) | 6,361 (77.1%) | 2,025 (65.8%) | 82  (72.6%) | <11 |
|  | Uninsured | 810  (6.8%) | 114 (10.4%) | 291  (7.7%) | 405  (5.8%) | 466  (8.0%) | 344  (5.6%) | 54  (30.2%) | 486  (5.9%) | 253  (8.2%) | <11 | <11 |
|  | Unknown | 1,088  (9.1%) | 107  (9.8%) | 328  (8.7%) | 653  (9.3%) | 598  (10.3%) | 490  (8.0%) | 14  (7.8%) | 613  (7.4%) | 200  (6.5%) | 12  (10.6%) | <11 |
| Alaska | Total | 1,478 | 166 | 462 | 850 | 654 | 824 | 36 | 1,033 | 41 | 97 | 260 |
|  | Medicaid | 320  (21.7%) | 37  (22.3%) | 94  (20.3%) | 189  (22.2%) | 143  (21.9%) | 177 (21.5%) | <11 | 186 (18.0%) | 12 (29.3%) | 25 (25.8%) | 87 (33.5%) |
|  | Other Insured | 958  (64.8%) | 98  (59.0%) | 307  (66.5%) | 553  (65.1%) | 407  (62.2%) | 551 (66.9%) | 20 (55.6%) | 687 (66.5%) | 27 (65.9%) | 55 (56.7%) | 168 (64.6%) |
|  | Uninsured | 49  (3.3%) | <11 | 15  (3.2%) | 24  (2.8%) | 24  (3.7%) | 25 (3.0%) | <11 | 35 (3.4%) | <11 | <11 | <11 |
|  | Unknown | 151  (10.2%) | 21  (12.7%) | 46  (10.0%) | 84  (9.9%) | 80  (12.2%) | 71 (8.6%) | <11 | 125 (12.1%) | <11 | <11 | <11 |
| Arizona | Total | 12,448 | 1,432 | 3,956 | 7,060 | 5,616 | 6,832 | 2,665 | 8,336 | 523 | 294 | 293 |
|  | Medicaid | 2,363 (19.0%) | 350 (24.4%) | 783 (19.8%) | 1,230 (17.4%) | 1,081 (19.2%) | 1,282 (18.8%) | 741 (27.8%) | 1,262 (15.1%) | 152 (29.1%) | 52 (17.7%) | 119 (40.6%) |
|  | Other Insured | 8,696 (69.9%) | 931 (65.0%) | 2,717 (68.7%) | 5,048 (71.5%) | 3,840 (68.4%) | 4,856 (71.1%) | 1,585 (59.5%) | 6,264 (75.1%) | 338 (64.6%) | 228 (77.6%) | 156 (53.2%) |
|  | Uninsured | 457 (3.7%) | 64 (4.5%) | 177 (4.5%) | 216 (3.1%) | 215 (3.8%) | 242 (3.5%) | 238 (8.9%) | 183 (2.2%) | 19 (3.6%) | <11 | <11 |
|  | Unknown | 932 (7.5%) | 87 (6.1%) | 279 (7.1%) | 566 (8.0%) | 480 (8.5%) | 452 (6.6%) | 101 (3.8%) | 627 (7.5%) | 14 (2.7%) | <11 | 17 (5.8%) |
| Arkansas | Total | 7,512 | 719 | 2,298 | 4,495 | 3,665 | 3,847 | 224 | 5,888 | 1,151 | 70 | 32 |
|  | Medicaid | 1,076 (14.3%) | 130 (18.1%) | 330 (14.4%) | 616 (13.7%) | 512 (14.0%) | 564 (14.7%) | 18 (8.0%) | 775 (13.2%) | 265 (23.0%) | <11 | <11 |
|  | Other Insured | 5,507 (73.3%) | 482 (67.0%) | 1,680 (73.1%) | 3,345 (74.4%) | 2,712 (74.0%) | 2,795 (72.7%) | 144 (64.3%) | 4,443 (75.5%) | 815 (70.8%) | 52 (74.3%) | 23 (71.9%) |
|  | Uninsured | 226 (3.0%) | 27 (3.8%) | 72 (3.1%) | 127 (2.8%) | 121 (3.3%) | 105 (2.7%) | 31 (13.8%) | 164 (2.8%) | 20 (1.7%) | <11 | <11 |
|  | Unknown | 703 (9.4%) | 80 (11.1%) | 216 (9.4%) | 407 (9.1%) | 320 (8.7%) | 383 (10.0%) | 31 (13.8%) | 506 (8.6%) | 51 (4.4%) | <11 | <11 |
| California | Total | 75,247 | 9,239 | 24,944 | 41,064 | 33,355 | 41,892 | 21,345 | 35,801 | 5,214 | 10,357 | 318 |
|  | Medicaid | 15,781 (21.0%) | 2,389 (25.9%) | 5,352 (21.5%) | 8,040 (19.6%) | 6,856 (20.6%) | 8,925 (21.3%) | 7,356 (34.5%) | 4,842 (13.5%) | 1,437 (27.6%) | 1,828 (17.6%) | 73 (23.0%) |
|  | Other Insured | 55,106 (73.2%) | 6,396 (69.2%) | 18,326 (73.5%) | 30,384 (74.0%) | 24,072 (72.2%) | 31,034 (74.1%) | 12,950 (60.7%) | 29,350 (82.0%) | 3,601 (69.1%) | 8,247 (79.6%) | 234 (73.6%) |
|  | Uninsured | 929 (1.2%) | 109 (1.2%) | 344 (1.4%) | 476 (1.2%) | 448 (1.3%) | 481 (1.1%) | 386 (1.8%) | 346 (1.0%) | 60 (1.2%) | 107 (1.0%) | <11 |
|  | Unknown | 3,431 (4.6%) | 345 (3.7%) | 922 (3.7%) | 2,164 (5.3%) | 1,979 (5.9%) | 1,452 (3.5%) | 653 (3.1%) | 1,263 (3.5%) | 116 (2.2%) | 175 (1.7%) | <11 |
| Colorado | Total | 10,764 | 1,309 | 3,561 | 5,894 | 4,935 | 5,829 | 1,634 | 8,185 | 462 | 245 | 76 |
|  | Medicaid | 1,908 (17.7%) | 288 (22.0%) | 613 (17.2%) | 1,007 (17.1%) | 846 (17.1%) | 1,062 (18.2%) | 508 (31.1%) | 1,154 (14.1%) | 149 (32.3%) | 42 (17.1%) | 24 (31.6%) |
|  | Other Insured | 8,145 (75.7%) | 929 (71.0%) | 2,703 (75.9%) | 4,513 (76.6%) | 3,707 (75.1%) | 4,438 (76.1%) | 918 (56.2%) | 6,613 (80.8%) | 294 (63.6%) | 188 (76.7%) | 46 (60.5%) |
|  | Uninsured | 348 (3.2%) | 59 (4.5%) | 126 (3.5%) | 163 (2.8%) | 176 (3.6%) | 172 (3.0%) | 158 (9.7%) | 167 (2.0%) | <11 | <11 | <11 |
|  | Unknown | 363 (3.4%) | 33 (2.5%) | 119 (3.3%) | 211 (3.6%) | 206 (4.2%) | 157 (2.7%) | 50 (3.1%) | 251 (3.1%) | <11 | <11 | <11 |
| Connecticut | Total | 8,993 | 805 | 2,846 | 5,342 | 4,219 | 4,774 | 1,060 | 6,626 | 924 | 219 | <11 |
|  | Medicaid | 1,700 (18.9%) | 207 (25.7%) | 564 (19.8%) | 929 (17.4%) | 778 (18.4%) | 922 (19.3%) | 402 (37.9%) | 940 (14.2%) | 293 (31.7%) | 49 (22.4%) | <11 |
|  | Other Insured | 6,526 (72.6%) | 502 (62.4%) | 2,046 (71.9%) | 3,978 (74.5%) | 3,046 (72.2%) | 3,480 (72.9%) | 518 (48.9%) | 5,233 (79.0%) | 531 (57.5%) | 156 (71.2%) | <11 |
|  | Uninsured | 215 (2.4%) | 42 (5.2%) | 66 (2.3%) | 107 (2.0%) | 109 (2.6%) | 106 (2.2%) | 78 (7.4%) | 83 (1.3%) | 42 (4.5%) | <11 | <11 |
|  | Unknown | 552 (6.1%) | 54 (6.7%) | 170 (6.0%) | 328 (6.1%) | 286 (6.8%) | 266 (5.6%) | 62 (5.8%) | 370 (5.6%) | 58 (6.3%) | <11 | <11 |
| Delaware | Total | 2,434 | 219 | 732 | 1,483 | 1,159 | 1,275 | 117 | 1,681 | 486 | 51 | <11 |
|  | Medicaid | 201 (8.3%) | 23 (10.5%) | 60 (8.2%) | 118 (8.0%) | 102 (8.8%) | 99 (7.8%) | 22 (18.8%) | 130 (7.7%) | 47 (9.7%) | <11 | <11 |
|  | Other Insured | 1,873 (77.0%) | 164 (74.9%) | 574 (78.4%) | 1,135 (76.5%) | 829 (71.5%) | 1,044 (81.9%) | 72 (61.5%) | 1,351 (80.4%) | 385 (79.2%) | 46 (90.2%) | <11 |
|  | Uninsured | 43 (1.8%) | <11 | 18 (2.5%) | 19 (1.3%) | 17 (1.5%) | 26 (2.0%) | <11 | 24 (1.4%) | <11 | <11 | <11 |
|  | Unknown | 317 (13.0%) | 26 (11.9%) | 80 (10.9%) | 211 (14.2%) | 211 (18.2%) | 106 (8.3%) | 15 (12.8%) | 176 (10.5%) | 46 (9.5%) | <11 | <11 |
| District of Columbia | Total | 1,331 | 200 | 406 | 725 | 606 | 725 | 81 | 411 | 764 | 33 | <11 |
|  | Medicaid | 466 (35.0%) | 56 (28.0%) | 128 (31.5%) | 282 (38.9%) | 204 (33.7%) | 262 (36.1%) | 40 (49.4%) | 21 (5.1%) | 394 (51.6%) | <11 | <11 |
|  | Other Insured | 753 (56.6%) | 127 (63.5%) | 247 (60.8%) | 379 (52.3%) | 339 (55.9%) | 414 (57.1%) | 38 (46.9%) | 348 (84.7%) | 336 (44.0%) | 22 (66.7%) | <11 |
|  | Uninsured | 12 (0.9%) | <11 | <11 | <11 | <11 | <11 | <11 | <11 | <11 | <11 | <11 |
|  | Unknown | 100 (7.5%) | 13 (6.5%) | 28 (6.9%) | 59 (8.1%) | 55 (9.1%) | 45 (6.2%) | <11 | 38 (9.2%) | 28 (3.7%) | <11 | <11 |
| Florida | Total | 49,231 | 4,686 | 15,459 | 29,086 | 22,416 | 26,815 | 9,772 | 30,755 | 6,545 | 890 | 85 |
|  | Medicaid | 5,278 (10.7%) | 604 (12.9%) | 1,715 (11.1%) | 2,959 (10.2%) | 2,462 (11.0%) | 2,816 (10.5%) | 1,173 (12.0%) | 2,866 (9.3%) | 1,131 (17.3%) | 42 (4.7%) | 11 (12.9%) |
|  | Other Insured | 35,498 (72.1%) | 3,100 (66.2%) | 11,041 (71.4%) | 21,357 (73.4%) | 15,829 (70.6%) | 19,669 (73.4%) | 7,200 (73.7%) | 22,577 (73.4%) | 4,436 (67.8%) | 769 (86.4%) | 67 (78.8%) |
|  | Uninsured | 2,788 (5.7%) | 344 (7.3%) | 983 (6.4%) | 1,461 (5.0%) | 1,422 (6.3%) | 1,366 (5.1%) | 674 (6.9%) | 1,599 (5.2%) | 435 (6.6%) | 34 (3.8%) | <11 |
|  | Unknown | 5,667 (11.5%) | 638 (13.6%) | 1,720 (11.1%) | 3,309 (11.4%) | 2,703 (12.1%) | 2,964 (11.1%) | 725 (7.4%) | 3,713 (12.1%) | 543 (8.3%) | 45 (5.1%) | <11 |
| Georgia | Total | 24,592 | 2,522 | 8,117 | 13,953 | 11,871 | 12,721 | 1,302 | 14,665 | 7,774 | 702 | 25 |
|  | Medicaid | 2,945 (12.0%) | 352 (14.0%) | 863 (10.6%) | 1,730 (12.4%) | 1,253 (10.6%) | 1,692 (13.3%) | 142 (10.9%) | 1,324 (9.0%) | 1,399 (18.0%) | 70 (10.0%) | <11 |
|  | Other Insured | 16,601 (67.5%) | 1,627 (64.5%) | 5,596 (68.9%) | 9,378 (67.2%) | 7,713 (65.0%) | 8,888 (69.9%) | 693 (53.2%) | 10,375 (70.7%) | 4,950 (63.7%) | 512 (72.9%) | 16 (64.0%) |
|  | Uninsured | 2,137 (8.7%) | 291 (11.5%) | 737 (9.1%) | 1,109 (7.9%) | 1,143 (9.6%) | 994 (7.8%) | 358 (27.5%) | 1,009 (6.9%) | 698 (9.0%) | 63 (9.0%) | <11 |
|  | Unknown | 2,909 (11.8%) | 252 (10.0%) | 921 (11.3%) | 1,736 (12.4%) | 1,762 (14.8%) | 1,147 (9.0%) | 109 (8.4%) | 1,957 (13.3%) | 727 (9.4%) | 57 (8.1%) | <11 |
| Hawaii | Total | 3,050 | 347 | 1,009 | 1,694 | 1,321 | 1,729 | 202 | 812 | 42 | 1,892 | 13 |
|  | Medicaid | 549 (18.0%) | 77 (22.2%) | 180 (17.8%) | 292 (17.2%) | 227 (17.2%) | 322 (18.6%) | 54 (26.7%) | 127 (15.6%) | 11 (26.2%) | 347 (18.3%) | <11 |
|  | Other Insured | 2,101 (68.9%) | 230 (66.3%) | 713 (70.7%) | 1,158 (68.4%) | 862 (65.3%) | 1,239 (71.7%) | 127 (62.9%) | 556 (68.5%) | 25 (59.5%) | 1,379 (72.9%) | <11 |
|  | Uninsured | 24 (0.8%) | <11 | 11 (1.1%) | <11 | 13 (1.0%) | 11 (0.6%) | <11 | <11 | <11 | 12 (0.6%) | <11 |
|  | Unknown | 376 (12.3%) | 37 (10.7%) | 105 (10.4%) | 234 (13.8%) | 219 (16.6%) | 157 (9.1%) | 19 (9.4%) | 122 (15.0%) | <11 | 154 (8.1%) | <11 |
| Idaho | Total | 3,522 | 417 | 1,048 | 2,057 | 1,694 | 1,828 | 245 | 3,137 | 19 | 39 | 25 |
|  | Medicaid | 427 (12.1%) | 60 (14.4%) | 134 (12.8%) | 233 (11.3%) | 186 (11.0%) | 241 (13.2%) | 47 (19.2%) | 357 (11.4%) | <11 | <11 | <11 |
|  | Other Insured | 2,528 (71.8%) | 269 (64.5%) | 762 (72.7%) | 1,497 (72.8%) | 1,194 (70.5%) | 1,334 (73.0%) | 150 (61.2%) | 2,300 (73.3%) | 14 (73.7%) | 31 (79.5%) | 16 (64.0%) |
|  | Uninsured | 228 (6.5%) | 41 (9.8%) | 69 (6.6%) | 118 (5.7%) | 111 (6.6%) | 117 (6.4%) | 35 (14.3%) | 190 (6.1%) | <11 | <11 | <11 |
|  | Unknown | 339 (9.6%) | 47 (11.3%) | 83 (7.9%) | 209 (10.2%) | 203 (12.0%) | 136 (7.4%) | 13 (5.3%) | 290 (9.2%) | <11 | <11 | <11 |
| Illinois | Total | 29,628 | 3,017 | 9,348 | 17,263 | 13,844 | 15,784 | 3,187 | 20,411 | 4,497 | 1,039 | 45 |
|  | Medicaid | 4,873 (16.4%) | 541 (17.9%) | 1,430 (15.3%) | 2,902 (16.8%) | 2,344 (16.9%) | 2,529 (16.0%) | 607 (19.0%) | 2,549 (12.5%) | 1,516 (33.7%) | 147 (14.1%) | 12 (26.7%) |
|  | Other Insured | 22,316 (75.3%) | 2,172 (72.0%) | 7,069 (75.6%) | 13,075 (75.7%) | 10,194 (73.6%) | 12,122 (76.8%) | 2,031 (63.7%) | 16,537 (81.0%) | 2,700 (60.0%) | 775 (74.6%) | 28 (62.2%) |
|  | Uninsured | 1,094 (3.7%) | 126 (4.2%) | 434 (4.6%) | 534 (3.1%) | 548 (4.0%) | 546 (3.5%) | 446 (14.0%) | 393 (1.9%) | 151 (3.4%) | 87 (8.4%) | <11 |
|  | Unknown | 1,345 (4.5%) | 178 (5.9%) | 415 (4.4%) | 752 (4.4%) | 758 (5.5%) | 587 (3.7%) | 103 (3.2%) | 932 (4.6%) | 130 (2.9%) | 30 (2.9%) | <11 |
| Indiana | Total | 14,226 | 1,432 | 4,374 | 8,420 | 6,593 | 7,633 | 536 | 12,156 | 1,197 | 174 | 11 |
|  | Medicaid | 2,331 (16.4%) | 282 (19.7%) | 688 (15.7%) | 1,361 (16.2%) | 1,021 (15.5%) | 1,310 (17.2%) | 142 (26.5%) | 1,792 (14.7%) | 344 (28.7%) | 26 (14.9%) | <11 |
|  | Other Insured | 10,996 (77.3%) | 1,049 (73.3%) | 3,371 (77.1%) | 6,576 (78.1%) | 5,108 (77.5%) | 5,888 (77.1%) | 321 (59.9%) | 9,653 (79.4%) | 771 (64.4%) | 139 (79.9%) | <11 |
|  | Uninsured | 503 (3.5%) | 68 (4.7%) | 190 (4.3%) | 245 (2.9%) | 257 (3.9%) | 246 (3.2%) | 45 (8.4%) | 406 (3.3%) | 38 (3.2%) | <11 | <11 |
|  | Unknown | 396 (2.8%) | 33 (2.3%) | 125 (2.9%) | 238 (2.8%) | 207 (3.1%) | 189 (2.5%) | 28 (5.2%) | 305 (2.5%) | 44 (3.7%) | <11 | <11 |
| Iowa | Total | 7,680 | 815 | 2,302 | 4,563 | 3,697 | 3,983 | 180 | 7,065 | 253 | 96 | 14 |
|  | Medicaid | 1,149 (15.0%) | 160 (19.6%) | 353 (15.3%) | 636 (13.9%) | 548 (14.8%) | 601 (15.1%) | 55 (30.6%) | 962 (13.6%) | 101 (39.9%) | 18 (18.8%) | <11 |
|  | Other Insured | 5,961 (77.6%) | 567 (69.6%) | 1,772 (77.0%) | 3,622 (79.4%) | 2,865 (77.5%) | 3,096 (77.7%) | 99 (55.0%) | 5,631 (79.7%) | 137 (54.2%) | 71 (74.0%) | <11 |
|  | Uninsured | 107 (1.4%) | 18 (2.2%) | 31 (1.3%) | 58 (1.3%) | 50 (1.4%) | 57 (1.4%) | 15 (8.3%) | 82 (1.2%) | <11 | <11 | <11 |
|  | Unknown | 463 (6.0%) | 70 (8.6%) | 146 (6.3%) | 247 (5.4%) | 234 (6.3%) | 229 (5.7%) | 11 (6.1%) | 390 (5.5%) | <11 | <11 | <11 |
| Kentucky | Total | 12,346 | 1,149 | 3,946 | 7,251 | 5,939 | 6,407 | 180 | 10,957 | 945 | 83 | <11 |
|  | Medicaid | 3,058 (24.8%) | 336 (29.2%) | 1,059 (26.8%) | 1,663 (22.9%) | 1,363 (22.9%) | 1,695 (26.5%) | 51 (28.3%) | 2,676 (24.4%) | 298 (31.5%) | 22 (26.5%) | <11 |
|  | Other Insured | 8,502 (68.9%) | 738 (64.2%) | 2,652 (67.2%) | 5,112 (70.5%) | 4,124 (69.4%) | 4,378 (68.3%) | 88 (48.9%) | 7,696 (70.2%) | 603 (63.8%) | 59 (71.1%) | <11 |
|  | Uninsured | 206 (1.7%) | 21 (1.8%) | 74 (1.9%) | 111 (1.5%) | 99 (1.7%) | 107 (1.7%) | 34 (18.9%) | 150 (1.4%) | 18 (1.9%) | <11 | <11 |
|  | Unknown | 580 (4.7%) | 54 (4.7%) | 161 (4.1%) | 365 (5.0%) | 353 (5.9%) | 227 (3.5%) | <11 | 435 (4.0%) | 26 (2.8%) | <11 | <11 |
| Louisiana | Total | 12,099 | 1,208 | 3,664 | 7,227 | 6,195 | 5,904 | 322 | 7,650 | 3,858 | 139 | 22 |
|  | Medicaid | 3,215 (26.6%) | 374 (31.0%) | 977 (26.7%) | 1,864 (25.8%) | 1,516 (24.5%) | 1,699 (28.8%) | 76 (23.6%) | 1,527 (20.0%) | 1,556 (40.3%) | 41 (29.5%) | <11 |
|  | Other Insured | 8,005 (66.2%) | 714 (59.1%) | 2,409 (65.7%) | 4,882 (67.6%) | 4,194 (67.7%) | 3,811 (64.5%) | 162 (50.3%) | 5,608 (73.3%) | 2,085 (54.0%) | 80 (57.6%) | 18 (81.8%) |
|  | Uninsured | 436 (3.6%) | 66 (5.5%) | 144 (3.9%) | 226 (3.1%) | 230 (3.7%) | 206 (3.5%) | 66 (20.5%) | 215 (2.8%) | 135 (3.5%) | 14 (10.1%) | <11 |
|  | Unknown | 443 (3.7%) | 54 (4.5%) | 134 (3.7%) | 255 (3.5%) | 255 (4.1%) | 188 (3.2%) | 18 (5.6%) | 300 (3.9%) | 82 (2.1%) | <11 | <11 |
| Maine | Total | 3,697 | 309 | 1,059 | 2,329 | 1,731 | 1,966 | 25 | 3,551 | 36 | 22 | 23 |
|  | Medicaid | 791 (21.4%) | 70 (22.7%) | 235 (22.2%) | 486 (20.9%) | 374 (21.6%) | 417 (21.2%) | <11 | 756 (21.3%) | 13 (36.1%) | <11 | 12 (52.2%) |
|  | Other Insured | 2,609 (70.6%) | 207 (67.0%) | 744 (70.3%) | 1,658 (71.2%) | 1,229 (71.0%) | 1,380 (70.2%) | 20 (80.0%) | 2,539 (71.5%) | 14 (38.9%) | 14 (63.6%) | <11 |
|  | Uninsured | 154 (4.2%) | 16 (5.2%) | 44 (4.2%) | 94 (4.0%) | 71 (4.1%) | 83 (4.2%) | <11 | 141 (4.0%) | <11 | <11 | <11 |
|  | Unknown | 143 (3.9%) | 16 (5.2%) | 36 (3.4%) | 91 (3.9%) | 57 (3.3%) | 86 (4.4%) | <11 | 115 (3.2%) | <11 | <11 | <11 |
| Maryland | Total | 14,689 | 1,414 | 4,659 | 8,616 | 6,807 | 7,882 | 747 | 8,496 | 4,620 | 690 | 12 |
|  | Medicaid | 1,732 (11.8%) | 210 (14.9%) | 530 (11.4%) | 992 (11.5%) | 794 (11.7%) | 938 (11.9%) | 114 (15.3%) | 737 (8.7%) | 819 (17.7%) | 56 (8.1%) | <11 |
|  | Other Insured | 11,510 (78.4%) | 1,044 (73.8%) | 3,694 (79.3%) | 6,772 (78.6%) | 5,232 (76.9%) | 6,278 (79.6%) | 498 (66.7%) | 6,925 (81.5%) | 3,440 (74.5%) | 586 (84.9%) | <11 |
|  | Uninsured | 227 (1.5%) | 44 (3.1%) | 74 (1.6%) | 109 (1.3%) | 123 (1.8%) | 104 (1.3%) | 71 (9.5%) | 77 (0.9%) | 62 (1.3%) | 12 (1.7%) | <11 |
|  | Unknown | 1,220 (8.3%) | 116 (8.2%) | 361 (7.7%) | 743 (8.6%) | 658 (9.7%) | 562 (7.1%) | 64 (8.6%) | 757 (8.9%) | 299 (6.5%) | 36 (5.2%) | <11 |
| Massachusetts | Total | 15,500 | 1,470 | 4,959 | 9,071 | 7,080 | 8,420 | 1,105 | 12,243 | 1,084 | 622 | 20 |
|  | Medicaid | 2,854 (18.4%) | 286 (19.5%) | 872 (17.6%) | 1,696 (18.7%) | 1,341 (18.9%) | 1,513 (18.0%) | 468 (42.4%) | 1,902 (15.5%) | 282 (26.0%) | 96 (15.4%) | <11 |
|  | Other Insured | 12,089 (78.0%) | 1,141 (77.6%) | 3,942 (79.5%) | 7,006 (77.2%) | 5,421 (76.6%) | 6,668 (79.2%) | 595 (53.8%) | 9,929 (81.1%) | 738 (68.1%) | 512 (82.3%) | 15 (75.0%) |
|  | Uninsured | 70 (0.5%) | 14 (1.0%) | 21 (0.4%) | 35 (0.4%) | 38 (0.5%) | 32 (0.4%) | <11 | 40 (0.3%) | 12 (1.1%) | <11 | <11 |
|  | Unknown | 487 (3.1%) | 29 (2.0%) | 124 (2.5%) | 334 (3.7%) | 280 (4.0%) | 207 (2.5%) | 32 (2.9%) | 372 (3.0%) | 52 (4.8%) | 11 (1.8%) | <11 |
| Michigan | Total | 23,171 | 2,060 | 7,185 | 13,926 | 11,204 | 11,967 | 502 | 18,409 | 3,082 | 387 | 107 |
|  | Medicaid | 3,619 (15.6%) | 406 (19.7%) | 1,175 (16.4%) | 2,038 (14.6%) | 1,713 (15.3%) | 1,906 (15.9%) | 136 (27.1%) | 2,700 (14.7%) | 652 (21.2%) | 51 (13.2%) | 25 (23.4%) |
|  | Other Insured | 14,310 (61.8%) | 1,193 (57.9%) | 4,369 (60.8%) | 8,748 (62.8%) | 6,926 (61.8%) | 7,384 (61.7%) | 244 (48.6%) | 12,236 (66.5%) | 1,266 (41.1%) | 232 (59.9%) | 66 (61.7%) |
|  | Uninsured | 254 (1.1%) | 27 (1.3%) | 85 (1.2%) | 142 (1.0%) | 134 (1.2%) | 120 (1.0%) | <11 | 197 (1.1%) | 35 (1.1%) | <11 | <11 |
|  | Unknown | 4,988 (21.5%) | 434 (21.1%) | 1,556 (21.7%) | 2,998 (21.5%) | 2,431 (21.7%) | 2,557 (21.4%) | 112 (22.3%) | 3,276 (17.8%) | 1,129 (36.6%) | 101 (26.1%) | 15 (14.0%) |
| Mississippi | Total | 7,413 | 629 | 2,266 | 4,518 | 3,712 | 3,701 | 83 | 4,400 | 2,838 | 61 | 18 |
|  | Medicaid | 1,464 (19.7%) | 140 (22.3%) | 410 (18.1%) | 914 (20.2%) | 743 (20.0%) | 721 (19.5%) | 11 (13.3%) | 621 (14.1%) | 819 (28.9%) | <11 | <11 |
|  | Other Insured | 5,262 (71.0%) | 422 (67.1%) | 1,625 (71.7%) | 3,215 (71.2%) | 2,595 (69.9%) | 2,667 (72.1%) | 47 (56.6%) | 3,379 (76.8%) | 1,767 (62.3%) | 45 (73.8%) | 16 (88.9%) |
|  | Uninsured | 549 (7.4%) | 56 (8.9%) | 193 (8.5%) | 300 (6.6%) | 293 (7.9%) | 256 (6.9%) | 24 (28.9%) | 300 (6.8%) | 217 (7.6%) | <11 | <11 |
|  | Unknown | 138 (1.9%) | 11 (1.7%) | 38 (1.7%) | 89 (2.0%) | 81 (2.2%) | 57 (1.5%) | <11 | 100 (2.3%) | 35 (1.2%) | <11 | <11 |
| Missouri | Total | 14,499 | 1,411 | 4,497 | 8,591 | 6,702 | 7,797 | 266 | 12,116 | 1,792 | 170 | 17 |
|  | Medicaid | 2,274 (15.7%) | 232 (16.4%) | 683 (15.2%) | 1,359 (15.8%) | 1,058 (15.8%) | 1,216 (15.6%) | 47 (17.7%) | 1,663 (13.7%) | 522 (29.1%) | 23 (13.5%) | <11 |
|  | Other Insured | 10,908 (75.2%) | 1,018 (72.1%) | 3,431 (76.3%) | 6,459 (75.2%) | 4,956 (73.9%) | 5,952 (76.3%) | 143 (53.8%) | 9,455 (78.0%) | 1,076 (60.0%) | 131 (77.1%) | <11 |
|  | Uninsured | 794 (5.5%) | 125 (8.9%) | 250 (5.6%) | 419 (4.9%) | 400 (6.0%) | 394 (5.1%) | 70 (26.3%) | 559 (4.6%) | 151 (8.4%) | 11 (6.5%) | <11 |
|  | Unknown | 523 (3.6%) | 36 (2.6%) | 133 (3.0%) | 354 (4.1%) | 288 (4.3%) | 235 (3.0%) | <11 | 439 (3.6%) | 43 (2.4%) | <11 | <11 |
| Montana | Total | 2,455 | 264 | 686 | 1,505 | 1,176 | 1,279 | 32 | 2,202 | 14 | 13 | 119 |
|  | Medicaid | 514 (20.9%) | 64 (24.2%) | 160 (23.3%) | 290 (19.3%) | 235 (20.0%) | 279 (21.8%) | <11 | 431 (19.6%) | <11 | <11 | 54 (45.4%) |
|  | Other Insured | 1,749 (71.2%) | 176 (66.7%) | 479 (69.8%) | 1,094 (72.7%) | 833 (70.8%) | 916 (71.6%) | 22 (68.8%) | 1,620 (73.6%) | <11 | <11 | 55 (46.2%) |
|  | Uninsured | 84 (3.4%) | 12 (4.5%) | 22 (3.2%) | 50 (3.3%) | 45 (3.8%) | 39 (3.0%) | <11 | 76 (3.5%) | <11 | <11 | <11 |
|  | Unknown | 108 (4.4%) | 12 (4.5%) | 25 (3.6%) | 71 (4.7%) | 63 (5.4%) | 45 (3.5%) | <11 | 75 (3.4%) | <11 | <11 | <11 |
| Nebraska | Total | 4,300 | 530 | 1,251 | 2,519 | 2,042 | 2,258 | 248 | 3,654 | 175 | 62 | 33 |
|  | Medicaid | 288 (6.7%) | 41 (7.7%) | 89 (7.1%) | 158 (6.3%) | 133 (6.5%) | 155 (6.9%) | 25 (10.1%) | 220 (6.0%) | 18 (10.3%) | <11 | 12 (36.4%) |
|  | Other Insured | 3,176 (73.9%) | 361 (68.1%) | 923 (73.8%) | 1,892 (75.1%) | 1,450 (71.0%) | 1,726 (76.4%) | 138 (55.6%) | 2,809 (76.9%) | 133 (76.0%) | 47 (75.8%) | 16 (48.5%) |
|  | Uninsured | 202 (4.7%) | 33 (6.2%) | 68 (5.4%) | 101 (4.0%) | 104 (5.1%) | 98 (4.3%) | 43 (17.3%) | 134 (3.7%) | 16 (9.1%) | <11 | <11 |
|  | Unknown | 634 (14.7%) | 95 (17.9%) | 171 (13.7%) | 368 (14.6%) | 355 (17.4%) | 279 (12.4%) | 42 (16.9%) | 491 (13.4%) | <11 | <11 | <11 |
| New Hampshire | Total | 3,626 | 309 | 1,045 | 2,272 | 1,727 | 1,899 | 81 | 3,390 | 32 | 39 | <11 |
|  | Medicaid | 418 (11.5%) | 46 (14.9%) | 111 (10.6%) | 261 (11.5%) | 182 (10.5%) | 236 (12.4%) | 17 (21.0%) | 384 (11.3%) | <11 | <11 | <11 |
|  | Other Insured | 2,926 (80.7%) | 247 (79.9%) | 852 (81.5%) | 1,827 (80.4%) | 1,378 (79.8%) | 1,548 (81.5%) | 57 (70.4%) | 2,778 (81.9%) | 22 (68.8%) | 31 (79.5%) | <11 |
|  | Uninsured | 91 (2.5%) | <11 | 32 (3.1%) | 54 (2.4%) | 58 (3.4%) | 33 (1.7%) | <11 | 85 (2.5%) | <11 | <11 | <11 |
|  | Unknown | 191 (5.3%) | 11 (3.6%) | 50 (4.8%) | 130 (5.7%) | 109 (6.3%) | 82 (4.3%) | <11 | 143 (4.2%) | <11 | <11 | <11 |
| New Jersey | Total | 22,734 | 2,245 | 7,412 | 13,077 | 10,814 | 11,920 | 3,206 | 14,335 | 2,963 | 1,412 | 25 |
|  | Medicaid | 2,452 (10.8%) | 322 (14.3%) | 813 (11.0%) | 1,317 (10.1%) | 1,137 (10.5%) | 1,315 (11.0%) | 605 (18.9%) | 1,070 (7.5%) | 614 (20.7%) | 132 (9.3%) | <11 |
|  | Other Insured | 16,999 (74.8%) | 1,589 (70.8%) | 5,619 (75.8%) | 9,791 (74.9%) | 7,702 (71.2%) | 9,297 (78.0%) | 1,948 (60.8%) | 11,790 (82.2%) | 1,931 (65.2%) | 1,110 (78.6%) | 15 (60.0%) |
|  | Uninsured | 836 (3.7%) | 143 (6.4%) | 304 (4.1%) | 389 (3.0%) | 362 (3.3%) | 474 (4.0%) | 373 (11.6%) | 255 (1.8%) | 126 (4.3%) | 69 (4.9%) | <11 |
|  | Unknown | 2,447 (10.8%) | 191 (8.5%) | 676 (9.1%) | 1,580 (12.1%) | 1,613 (14.9%) | 834 (7.0%) | 280 (8.7%) | 1,220 (8.5%) | 292 (9.9%) | 101 (7.2%) | <11 |
| New Mexico | Total | 4,046 | 457 | 1,284 | 2,305 | 1,797 | 2,249 | 1,706 | 1,844 | 69 | 69 | 226 |
|  | Medicaid | 875 (21.6%) | 145 (31.7%) | 292 (22.7%) | 438 (19.0%) | 390 (21.7%) | 485 (21.6%) | 411 (24.1%) | 325 (17.6%) | 22 (31.9%) | 15 (21.7%) | 91 (40.3%) |
|  | Other Insured | 2,423 (59.9%) | 225 (49.2%) | 740 (57.6%) | 1,458 (63.3%) | 1,017 (56.6%) | 1,406 (62.5%) | 995 (58.3%) | 1,210 (65.6%) | 36 (52.2%) | 40 (58.0%) | 114 (50.4%) |
|  | Uninsured | 138 (3.4%) | 25 (5.5%) | 62 (4.8%) | 51 (2.2%) | 67 (3.7%) | 71 (3.2%) | 99 (5.8%) | 32 (1.7%) | <11 | <11 | <11 |
|  | Unknown | 610 (15.1%) | 62 (13.6%) | 190 (14.8%) | 358 (15.5%) | 323 (18.0%) | 287 (12.8%) | 201 (11.8%) | 277 (15.0%) | 11 (15.9%) | 11 (15.9%) | 18 (8.0%) |
| New York | Total | 49,938 | 5,566 | 15,898 | 28,474 | 23,215 | 26,723 | 6,803 | 30,833 | 7,552 | 3,715 | 99 |
|  | Medicaid | 11,424 (22.9%) | 1,540 (27.7%) | 3,755 (23.6%) | 6,129 (21.5%) | 5,115 (22.0%) | 6,309 (23.6%) | 2,685 (39.5%) | 4,393 (14.2%) | 2,663 (35.3%) | 1,433 (38.6%) | 33 (33.3%) |
|  | Other Insured | 35,396 (70.9%) | 3,647 (65.5%) | 11,168 (70.2%) | 20,581 (72.3%) | 16,383 (70.6%) | 19,013 (71.1%) | 3,650 (53.7%) | 24,834 (80.5%) | 4,425 (58.6%) | 2,079 (56.0%) | 61 (61.6%) |
|  | Uninsured | 569 (1.1%) | 91 (1.6%) | 196 (1.2%) | 282 (1.0%) | 293 (1.3%) | 276 (1.0%) | 171 (2.5%) | 245 (0.8%) | 99 (1.3%) | 36 (1.0%) | <11 |
|  | Unknown | 2,549 (5.1%) | 288 (5.2%) | 779 (4.9%) | 1,482 (5.2%) | 1,424 (6.1%) | 1,125 (4.2%) | 297 (4.4%) | 1,361 (4.4%) | 365 (4.8%) | 167 (4.5%) | <11 |
| North Carolina | Total | 25,190 | 2,288 | 8,087 | 14,815 | 12,032 | 13,158 | 1,105 | 17,443 | 5,487 | 399 | 227 |
|  | Medicaid | 3,479 (13.8%) | 362 (15.8%) | 1,113 (13.8%) | 2,004 (13.5%) | 1,701 (14.1%) | 1,778 (13.5%) | 168 (15.2%) | 1,962 (11.2%) | 1,232 (22.5%) | 40 (10.0%) | 52 (22.9%) |
|  | Other Insured | 19,394 (77.0%) | 1,628 (71.2%) | 6,176 (76.4%) | 11,590 (78.2%) | 9,078 (75.4%) | 10,316 (78.4%) | 598 (54.1%) | 14,302 (82.0%) | 3,809 (69.4%) | 329 (82.5%) | 152 (67.0%) |
|  | Uninsured | 1,508 (6.0%) | 218 (9.5%) | 572 (7.1%) | 718 (4.8%) | 784 (6.5%) | 724 (5.5%) | 312 (28.2%) | 803 (4.6%) | 333 (6.1%) | 27 (6.8%) | 15 (6.6%) |
|  | Unknown | 809 (3.2%) | 80 (3.5%) | 226 (2.8%) | 503 (3.4%) | 469 (3.9%) | 340 (2.6%) | 27 (2.4%) | 376 (2.2%) | 113 (2.1%) | <11 | <11 |
| North Dakota | Total | 1,565 | 175 | 430 | 960 | 756 | 809 | 26 | 1,379 | 28 | 11 | 75 |
|  | Medicaid | 145 (9.3%) | 15 (8.6%) | 47 (10.9%) | 83 (8.6%) | 66 (8.7%) | 79 (9.8%) | <11 | 112 (8.1%) | <11 | <11 | 11 (14.7%) |
|  | Other Insured | 1,323 (84.5%) | 145 (82.9%) | 356 (82.8%) | 822 (85.6%) | 641 (84.8%) | 682 (84.3%) | 14 (53.8%) | 1,190 (86.3%) | 12 (42.9%) | <11 | 59 (78.7%) |
|  | Uninsured | 52 (3.3%) | 11 (6.3%) | 13 (3.0%) | 28 (2.9%) | 31 (4.1%) | 21 (2.6%) | <11 | 37 (2.7%) | <11 | <11 | <11 |
|  | Unknown | 45 (2.9%) | <11 | 14 (3.3%) | 27 (2.8%) | 18 (2.4%) | 27 (3.3%) | <11 | 40 (2.9%) | <11 | <11 | <11 |
| Ohio | Total | 29,523 | 2,865 | 8,936 | 17,722 | 14,024 | 15,499 | 449 | 24,718 | 3,420 | 343 | 30 |
|  | Medicaid | 5,663 (19.2%) | 721 (25.2%) | 1,763 (19.7%) | 3,179 (17.9%) | 2,549 (18.2%) | 3,114 (20.1%) | 132 (29.4%) | 4,145 (16.8%) | 1,248 (36.5%) | 72 (21.0%) | <11 |
|  | Other Insured | 22,298 (75.5%) | 1,989 (69.4%) | 6,740 (75.4%) | 13,569 (76.6%) | 10,608 (75.6%) | 11,690 (75.4%) | 246 (54.8%) | 19,480 (78.8%) | 2,022 (59.1%) | 251 (73.2%) | 23 (76.7%) |
|  | Uninsured | 736 (2.5%) | 87 (3.0%) | 229 (2.6%) | 420 (2.4%) | 376 (2.7%) | 360 (2.3%) | 53 (11.8%) | 574 (2.3%) | 72 (2.1%) | 18 (5.2%) | <11 |
|  | Unknown | 826 (2.8%) | 68 (2.4%) | 204 (2.3%) | 554 (3.1%) | 491 (3.5%) | 335 (2.2%) | 18 (4.0%) | 519 (2.1%) | 78 (2.3%) | <11 | <11 |
| Oklahoma | Total | 8,722 | 888 | 2,691 | 5,143 | 4,158 | 4,564 | 404 | 6,370 | 654 | 131 | 692 |
|  | Medicaid | 922 (10.6%) | 108 (12.2%) | 304 (11.3%) | 510 (9.9%) | 371 (8.9%) | 551 (12.1%) | 59 (14.6%) | 610 (9.6%) | 108 (16.5%) | 15 (11.5%) | 86 (12.4%) |
|  | Other Insured | 6,733 (77.2%) | 657 (74.0%) | 2,052 (76.3%) | 4,024 (78.2%) | 3,186 (76.6%) | 3,547 (77.7%) | 250 (61.9%) | 5,041 (79.1%) | 456 (69.7%) | 102 (77.9%) | 562 (81.2%) |
|  | Uninsured | 561 (6.4%) | 70 (7.9%) | 206 (7.7%) | 285 (5.5%) | 311 (7.5%) | 250 (5.5%) | 67 (16.6%) | 406 (6.4%) | 53 (8.1%) | <11 | 17 (2.5%) |
|  | Unknown | 506 (5.8%) | 53 (6.0%) | 129 (4.8%) | 324 (6.3%) | 290 (7.0%) | 216 (4.7%) | 28 (6.9%) | 313 (4.9%) | 37 (5.7%) | <11 | 27 (3.9%) |
| Oregon | Total | 8,605 | 988 | 2,693 | 4,924 | 3,856 | 4,749 | 647 | 7,219 | 174 | 338 | 133 |
|  | Medicaid | 1,644 (19.1%) | 223 (22.6%) | 569 (21.1%) | 852 (17.3%) | 781 (20.3%) | 863 (18.2%) | 209 (32.3%) | 1,251 (17.3%) | 66 (37.9%) | 61 (18.0%) | 39 (29.3%) |
|  | Other Insured | 6,439 (74.8%) | 718 (72.7%) | 1,952 (72.5%) | 3,769 (76.5%) | 2,824 (73.2%) | 3,615 (76.1%) | 386 (59.7%) | 5,566 (77.1%) | 96 (55.2%) | 260 (76.9%) | 89 (66.9%) |
|  | Uninsured | 159 (1.8%) | 14 (1.4%) | 67 (2.5%) | 78 (1.6%) | 71 (1.8%) | 88 (1.9%) | 28 (4.3%) | 112 (1.6%) | <11 | 12 (3.6%) | <11 |
|  | Unknown | 363 (4.2%) | 33 (3.3%) | 105 (3.9%) | 225 (4.6%) | 180 (4.7%) | 183 (3.9%) | 24 (3.7%) | 290 (4.0%) | <11 | <11 | <11 |
| Pennsylvania | Total | 31,662 | 2,974 | 9,524 | 19,164 | 15,056 | 16,606 | 1,368 | 25,768 | 3,253 | 598 | 32 |
|  | Medicaid | 4,627 (14.6%) | 594 (20.0%) | 1,518 (15.9%) | 2,515 (13.1%) | 2,059 (13.7%) | 2,568 (15.5%) | 482 (35.2%) | 3,023 (11.7%) | 947 (29.1%) | 117 (19.6%) | 14 (43.8%) |
|  | Other Insured | 25,323 (80.0%) | 2,243 (75.4%) | 7,533 (79.1%) | 15,547 (81.1%) | 12,009 (79.8%) | 13,314 (80.2%) | 766 (56.0%) | 21,702 (84.2%) | 2,107 (64.8%) | 459 (76.8%) | 17 (53.1%) |
|  | Uninsured | 439 (1.4%) | 51 (1.7%) | 141 (1.5%) | 247 (1.3%) | 227 (1.5%) | 212 (1.3%) | 62 (4.5%) | 295 (1.1%) | 55 (1.7%) | 13 (2.2%) | <11 |
|  | Unknown | 1,273 (4.0%) | 86 (2.9%) | 332 (3.5%) | 855 (4.5%) | 761 (5.1%) | 512 (3.1%) | 58 (4.2%) | 748 (2.9%) | 144 (4.4%) | <11 | <11 |
| Rhode Island | Total | 2,662 | 246 | 832 | 1,584 | 1,252 | 1,410 | 213 | 2,173 | 132 | 46 | <11 |
|  | Medicaid | 582 (21.9%) | 65 (26.4%) | 204 (24.5%) | 313 (19.8%) | 252 (20.1%) | 330 (23.4%) | 91 (42.7%) | 408 (18.8%) | 53 (40.2%) | 14 (30.4%) | <11 |
|  | Other Insured | 1,993 (74.9%) | 174 (70.7%) | 590 (70.9%) | 1,229 (77.6%) | 953 (76.1%) | 1,040 (73.8%) | 102 (47.9%) | 1,713 (78.8%) | 72 (54.5%) | 31 (67.4%) | <11 |
|  | Uninsured | 45 (1.7%) | <11 | 24 (2.9%) | 16 (1.0%) | 22 (1.8%) | 23 (1.6%) | 20 (9.4%) | 19 (0.9%) | <11 | <11 | <11 |
|  | Unknown | 42 (1.6%) | <11 | 14 (1.7%) | 26 (1.6%) | 25 (2.0%) | 17 (1.2%) | <11 | 33 (1.5%) | <11 | <11 | <11 |
| South California | Total | 11,475 | 1,064 | 3,692 | 6,719 | 5,463 | 6,012 | 326 | 7,845 | 2,934 | 125 | 36 |
|  | Medicaid | 1,287 (11.2%) | 141 (13.3%) | 437 (11.8%) | 709 (10.6%) | 572 (10.5%) | 715 (11.9%) | 47 (14.4%) | 693 (8.8%) | 527 (18.0%) | <11 | <11 |
|  | Other Insured | 8,262 (72.0%) | 708 (66.5%) | 2,648 (71.7%) | 4,906 (73.0%) | 3,809 (69.7%) | 4,453 (74.1%) | 172 (52.8%) | 6,012 (76.6%) | 1,908 (65.0%) | 109 (87.2%) | 28 (77.8%) |
|  | Uninsured | 817 (7.1%) | 102 (9.6%) | 295 (8.0%) | 420 (6.3%) | 452 (8.3%) | 365 (6.1%) | 76 (23.3%) | 482 (6.1%) | 245 (8.4%) | <11 | <11 |
|  | Unknown | 1,109 (9.7%) | 113 (10.6%) | 312 (8.5%) | 684 (10.2%) | 630 (11.5%) | 479 (8.0%) | 31 (9.5%) | 658 (8.4%) | 254 (8.7%) | <11 | <11 |
| South Dakota | Total | 1,822 | 203 | 538 | 1,081 | 875 | 947 | 36 | 1,611 | 19 | 14 | 133 |
|  | Medicaid | 135 (7.4%) | 24 (11.8%) | 34 (6.3%) | 77 (7.1%) | 52 (5.9%) | 83 (8.8%) | <11 | 87 (5.4%) | <11 | <11 | 36 (27.1%) |
|  | Other Insured | 1,575 (86.4%) | 162 (79.8%) | 461 (85.7%) | 952 (88.1%) | 759 (86.7%) | 816 (86.2%) | 20 (55.6%) | 1,434 (89.0%) | <11 | 11 (78.6%) | 91 (68.4%) |
|  | Uninsured | 77 (4.2%) | 13 (6.4%) | 30 (5.6%) | 34 (3.1%) | 43 (4.9%) | 34 (3.6%) | <11 | 59 (3.7%) | <11 | <11 | <11 |
|  | Unknown | 35 (1.9%) | <11 | 13 (2.4%) | 18 (1.7%) | 21 (2.4%) | 14 (1.5%) | <11 | 31 (1.9%) | <11 | <11 | <11 |
| Tennessee | Total | 16,207 | 1,472 | 5,140 | 9,595 | 7,832 | 8,375 | 347 | 12,685 | 2,536 | 159 | 15 |
|  | Medicaid | 1,654 (10.2%) | 221 (15.0%) | 543 (10.6%) | 890 (9.3%) | 690 (8.8%) | 964 (11.5%) | 30 (8.6%) | 1,178 (9.3%) | 417 (16.4%) | 17 (10.7%) | <11 |
|  | Other Insured | 11,835 (73.0%) | 976 (66.3%) | 3,697 (71.9%) | 7,162 (74.6%) | 5,633 (71.9%) | 6,202 (74.1%) | 164 (47.3%) | 9,755 (76.9%) | 1,733 (68.3%) | 115 (72.3%) | <11 |
|  | Uninsured | 1,236 (7.6%) | 133 (9.0%) | 435 (8.5%) | 668 (7.0%) | 686 (8.8%) | 550 (6.6%) | 123 (35.4%) | 854 (6.7%) | 233 (9.2%) | 14 (8.8%) | <11 |
|  | Unknown | 1,482 (9.1%) | 142 (9.6%) | 465 (9.0%) | 875 (9.1%) | 823 (10.5%) | 659 (7.9%) | 30 (8.6%) | 898 (7.1%) | 153 (6.0%) | 13 (8.2%) | <11 |
| Texas | Total | 55,565 | 6,804 | 18,772 | 29,989 | 25,780 | 29,785 | 15,470 | 29,481 | 7,642 | 1,958 | 215 |
|  | Medicaid | 4,605 (8.3%) | 682 (10.0%) | 1,499 (8.0%) | 2,424 (8.1%) | 1,951 (7.6%) | 2,654 (8.9%) | 1,746 (11.3%) | 1,705 (5.8%) | 1,055 (13.8%) | 74 (3.8%) | 14 (6.5%) |
|  | Other Insured | 40,549 (73.0%) | 4,592 (67.5%) | 13,644 (72.7%) | 22,313 (74.4%) | 18,537 (71.9%) | 22,012 (73.9%) | 9,620 (62.2%) | 23,790 (80.7%) | 5,139 (67.2%) | 1,575 (80.4%) | 167 (77.7%) |
|  | Uninsured | 6,890 (12.4%) | 1,111 (16.3%) | 2,557 (13.6%) | 3,222 (10.7%) | 3,294 (12.8%) | 3,596 (12.1%) | 3,374 (21.8%) | 2,245 (7.6%) | 1,026 (13.4%) | 201 (10.3%) | 25 (11.6%) |
|  | Unknown | 3,521 (6.3%) | 419 (6.2%) | 1,072 (5.7%) | 2,030 (6.8%) | 1,998 (7.8%) | 1,523 (5.1%) | 730 (4.7%) | 1,741 (5.9%) | 422 (5.5%) | 108 (5.5%) | <11 |
| Utah | Total | 5,329 | 942 | 1,812 | 2,575 | 2,379 | 2,950 | 571 | 4,410 | 57 | 145 | 39 |
|  | Medicaid | 379 (7.1%) | 74 (7.9%) | 126 (7.0%) | 179 (7.0%) | 155 (6.5%) | 224 (7.6%) | 79 (13.8%) | 268 (6.1%) | <11 | 14 (9.7%) | 11 (28.2%) |
|  | Other Insured | 3,783 (71.0%) | 661 (70.2%) | 1,299 (71.7%) | 1,823 (70.8%) | 1,622 (68.2%) | 2,161 (73.3%) | 313 (54.8%) | 3,293 (74.7%) | 34 (59.6%) | 107 (73.8%) | 24 (61.5%) |
|  | Uninsured | 237 (4.4%) | 41 (4.4%) | 97 (5.4%) | 99 (3.8%) | 93 (3.9%) | 144 (4.9%) | 98 (17.2%) | 124 (2.8%) | <11 | 11 (7.6%) | <11 |
|  | Unknown | 930 (17.5%) | 166 (17.6%) | 290 (16.0%) | 474 (18.4%) | 509 (21.4%) | 421 (14.3%) | 81 (14.2%) | 725 (16.4%) | 14 (24.6%) | 13 (9.0%) | <11 |
| Vermont | Total | 1,560 | 132 | 505 | 923 | 728 | 832 | 16 | 1,487 | 16 | 12 | <11 |
|  | Medicaid | 355 (22.8%) | 51 (38.6%) | 113 (22.4%) | 191 (20.7%) | 166 (22.8%) | 189 (22.7%) | <11 | 336 (22.6%) | <11 | <11 | <11 |
|  | Other Insured | 1,144 (73.3%) | 79 (59.8%) | 371 (73.5%) | 694 (75.2%) | 527 (72.4%) | 617 (74.2%) | <11 | 1,098 (73.8%) | <11 | <11 | <11 |
|  | Uninsured | 27 (1.7%) | <11 | <11 | 18 (2.0%) | 16 (2.2%) | 11 (1.3%) | <11 | 24 (1.6%) | <11 | <11 | <11 |
|  | Unknown | 34 (2.2%) | <11 | 13 (2.6%) | 20 (2.2%) | 19 (2.6%) | 15 (1.8%) | <11 | 29 (2.0%) | <11 | <11 | <11 |
| Virginia | Total | 17,859 | 1,860 | 5,714 | 10,285 | 8,374 | 9,485 | 831 | 12,055 | 3,707 | 768 | 53 |
|  | Medicaid | 2,224 (12.5%) | 283 (15.2%) | 690 (12.1%) | 1,251 (12.2%) | 1,029 (12.3%) | 1,195 (12.6%) | 90 (10.8%) | 1,248 (10.4%) | 779 (21.0%) | 67 (8.7%) | <11 |
|  | Other Insured | 13,844 (77.5%) | 1,334 (71.7%) | 4,461 (78.1%) | 8,049 (78.3%) | 6,336 (75.7%) | 7,508 (79.2%) | 511 (61.5%) | 9,812 (81.4%) | 2,593 (69.9%) | 622 (81.0%) | 45 (84.9%) |
|  | Uninsured | 829 (4.6%) | 140 (7.5%) | 264 (4.6%) | 425 (4.1%) | 414 (4.9%) | 415 (4.4%) | 196 (23.6%) | 387 (3.2%) | 183 (4.9%) | 31 (4.0%) | <11 |
|  | Unknown | 962 (5.4%) | 103 (5.5%) | 299 (5.2%) | 560 (5.4%) | 595 (7.1%) | 367 (3.9%) | 34 (4.1%) | 608 (5.0%) | 152 (4.1%) | 48 (6.3%) | <11 |
| Washington | Total | 15,660 | 1,916 | 4,911 | 8,833 | 7,021 | 8,639 | 1,032 | 11,978 | 694 | 1,314 | 280 |
|  | Medicaid | 2,738 (17.5%) | 407 (21.2%) | 859 (17.5%) | 1,472 (16.7%) | 1,219 (17.4%) | 1,519 (17.6%) | 327 (31.7%) | 1,847 (15.4%) | 203 (29.3%) | 236 (18.0%) | 98 (35.0%) |
|  | Other Insured | 10,958 (70.0%) | 1,269 (66.2%) | 3,431 (69.9%) | 6,258 (70.8%) | 4,786 (68.2%) | 6,172 (71.4%) | 534 (51.7%) | 8,761 (73.1%) | 427 (61.5%) | 978 (74.4%) | 152 (54.3%) |
|  | Uninsured | 300 (1.9%) | 42 (2.2%) | 103 (2.1%) | 155 (1.8%) | 137 (2.0%) | 163 (1.9%) | 82 (7.9%) | 163 (1.4%) | 17 (2.4%) | 26 (2.0%) | <11 |
|  | Unknown | 1,664 (10.6%) | 198 (10.3%) | 518 (10.5%) | 948 (10.7%) | 879 (12.5%) | 785 (9.1%) | 89 (8.6%) | 1,207 (10.1%) | 47 (6.8%) | 74 (5.6%) | 23 (8.2%) |
| West Virginia | Total | 5,031 | 428 | 1,581 | 3,022 | 2,348 | 2,683 | 20 | 4,796 | 171 | 12 | <11 |
|  | Medicaid | 1,187 (23.6%) | 128 (29.9%) | 396 (25.0%) | 663 (21.9%) | 548 (23.3%) | 639 (23.8%) | <11 | 1,116 (23.3%) | 55 (32.2%) | <11 | <11 |
|  | Other Insured | 3,590 (71.4%) | 280 (65.4%) | 1,095 (69.3%) | 2,215 (73.3%) | 1,686 (71.8%) | 1,904 (71.0%) | 12 (60.0%) | 3,443 (71.8%) | 113 (66.1%) | <11 | <11 |
|  | Uninsured | 128 (2.5%) | <11 | 46 (2.9%) | 73 (2.4%) | 58 (2.5%) | 70 (2.6%) | <11 | 122 (2.5%) | <11 | <11 | <11 |
|  | Unknown | 126 (2.5%) | 11 (2.6%) | 44 (2.8%) | 71 (2.3%) | 56 (2.4%) | 70 (2.6%) | <11 | 115 (2.4%) | <11 | <11 | <11 |
| Wisconsin | Total | 14,154 | 1,326 | 4,226 | 8,602 | 6,870 | 7,284 | 516 | 12,266 | 964 | 174 | 139 |
|  | Medicaid | 1,914 (13.5%) | 236 (17.8%) | 598 (14.2%) | 1,080 (12.6%) | 932 (13.6%) | 982 (13.5%) | 119 (23.1%) | 1,343 (10.9%) | 372 (38.6%) | 40 (23.0%) | 30 (21.6%) |
|  | Other Insured | 11,801 (83.4%) | 1,036 (78.1%) | 3,494 (82.7%) | 7,271 (84.5%) | 5,709 (83.1%) | 6,092 (83.6%) | 346 (67.1%) | 10,597 (86.4%) | 562 (58.3%) | 128 (73.6%) | 104 (74.8%) |
|  | Uninsured | 215 (1.5%) | 27 (2.0%) | 82 (1.9%) | 106 (1.2%) | 104 (1.5%) | 111 (1.5%) | 40 (7.8%) | 143 (1.2%) | 20 (2.1%) | <11 | <11 |
|  | Unknown | 224 (1.6%) | 27 (2.0%) | 52 (1.2%) | 145 (1.7%) | 125 (1.8%) | 99 (1.4%) | 11 (2.1%) | 183 (1.5%) | <11 | <11 | <11 |
| Wyoming | Total | 1,250 | 137 | 363 | 750 | 597 | 653 | 65 | 1,146 | <11 | <11 | 13 |
|  | Medicaid | 80 (6.4%) | 14 (10.2%) | 32 (8.8%) | 34 (4.5%) | 25 (4.2%) | 55 (8.4%) | <11 | 69 (6.0%) | <11 | <11 | <11 |
|  | Other Insured | 870 (69.6%) | 93 (67.9%) | 233 (64.2%) | 544 (72.5%) | 416 (69.7%) | 454 (69.5%) | 43 (66.2%) | 800 (69.8%) | <11 | <11 | <11 |
|  | Uninsured | 87 (7.0%) | <11 | 36 (9.9%) | 46 (6.1%) | 55 (9.2%) | 32 (4.9%) | <11 | 80 (7.0%) | <11 | <11 | <11 |
|  | Unknown | 213 (17.0%) | 25 (18.2%) | 62 (17.1%) | 126 (16.8%) | 101 (16.9%) | 112 (17.2%) | 13 (20.0%) | 197 (17.2%) | <11 | <11 | <11 |

Source: Authors’ analysis of Cancer Incidence in North America (CiNA) 2010-2019 compiled by the North American Association of Central Cancer Registries

Notes: Column percentage reported. Cases with unknown insurance status at diagnosis were included.

Figure S4. Percent Medicaid Insured Among Incident Cancer Cases Diagnosed at Ages 18-64 Years in 2019, Including Cases with Unknown Insurance Status, 2019





Source: Authors’ analysis of Cancer Incidence in North America (CiNA) 2010-2019 compiled by the North American Association of Central Cancer Registries

Notes: Cases with unknown insurance status at diagnosis were included.

Figure S5. Percent Uninsured Among Incident Cancer Cases Diagnosed at Ages 18-64 Years in 2019, Including Cases with Unknown Insurance Status, 2019



Source: Authors’ analysis of Cancer Incidence in North America (CiNA) 2010-2019 compiled by the North American Association of Central Cancer Registries

Notes: Cases with unknown insurance status at diagnosis were included.

Table S4. Demographic Characteristics By Known Insurance Status at Diagnosis

|  |  | **Known Insurance Status** | | **Unknown Insurance Status** | |
| --- | --- | --- | --- | --- | --- |
|  |  | **n** | **%** | **n** | **%** |
| Age Groups | 18-39 years | 655441 | 10.2% | 66025 | 9.7% |
|  | 40-54 years | 2211911 | 34.4% | 222455 | 32.8% |
|  | 55-64 years | 3564765 | 55.4% | 389019 | 57.4% |
| Sex | Male | 3010969 | 46.8% | 365355 | 53.9% |
|  | Female | 3421148 | 53.2% | 312144 | 46.1% |
| Race/Ethnicity | Hispanic | 666353 | 10.4% | 47559 | 7.0% |
|  | NH-White | 4578661 | 71.2% | 459583 | 67.8% |
|  | NH-Black | 840174 | 13.1% | 105065 | 15.5% |
|  | NH-API | 254531 | 4.0% | 15218 | 2.2% |
|  | NH-AIAN | 37365 | 0.6% | 3969 | 0.6% |
|  | Other/Unknown | 55033 | 0.9% | 46105 | 6.8% |

Figure S6. Percent with Medicaid Coverage and Uninsured Among Individuals Aged 18-64 Years Newly Diagnosed With Cancer by Age, Sex, Race/Ethnicity, 2010

Source: Authors’ analysis of Cancer Incidence in North America (CiNA) 2010-2019 compiled by the North American Association of Central Cancer Registries

Notes: (A) Medicaid (B) Uninsured. Percentages calculated excluding cancer cases with unknown insurance status.
